# Inner retinal injury in experimental glaucoma is prevented upon AAV mediated Shp2 silencing in a caveolin dependent manner

**DOI:** 10.7150/thno.55472

**Published:** 2021-04-15

**Authors:** Mojdeh Abbasi, Vivek K. Gupta, Nitin Chitranshi, Veer Gupta, Reza Ranjbaran, Rashi Rajput, Kanishka Pushpitha, Devaraj KB, Yuyi You, Ghasem Hosseini Salekdeh, Robert G. Parton, Mehdi Mirzaei, Stuart L. Graham

**Affiliations:** 1 Department of Clinical Medicine, Faculty of Medicine and Health Sciences, Macquarie University, North Ryde, Sydney, NSW 2109, Australia.; 2 School of Medicine, Deakin University, Melbourne, VIC, Australia.; 3 Diagnostic Laboratory Sciences and Technology Research Center, School of Paramedical Sciences, Shiraz University of Medical Sciences, Shiraz, Iran.; 4 Save Sight Institute, Sydney University, Sydney NSW 2000, Australia.; 5 Department of Molecular Systems Biology, Cell Science Research Center, Royan Institute for Stem Cell Biology and Technology, Tehran, Iran.; 6 Department of Molecular Sciences, Macquarie University, North Ryde, Sydney, NSW 2109, Australia.; 7 Institute for Molecular Bioscience and Centre for Microscopy and Microanalysis, The University of Queensland, Queensland 4072, Australia.; 8 Institute for Molecular Bioscience, The University of Queensland, Brisbane, QLD, Australia.

**Keywords:** Glaucoma, Retinal Ganglion cells, Shp2 phosphatase, Caveolin, TrkB.

## Abstract

SH2 domain containing tyrosine phosphatase 2 (Shp2; PTPN11) regulates several intracellular pathways downstream of multiple growth factor receptors. Our studies implicate that Shp2 interacts with Caveolin-1 (Cav-1) protein in retinal ganglion cells (RGCs) and negatively regulates BDNF/TrkB signaling. This study aimed to investigate the mechanisms underlying the protective effects of shp2 silencing in the RGCs in glaucomatous conditions.

**Methods:** Shp2 was silenced in the Cav-1 deficient mice and the age matched wildtype littermates using adeno-associated viral (AAV) constructs. Shp2 expression modulation was performed in an acute and a chronic mouse model of experimental glaucoma. AAV2 expressing Shp2 eGFP-shRNA under a strong synthetic CAG promoter was administered intravitreally in the animals' eyes. The contralateral eye received AAV-eGFP-scramble-shRNA as control. Animals with Shp2 downregulation were subjected to either microbead injections or acute ocular hypertension experimental paradigm. Changes in inner retinal function were evaluated by measuring positive scotopic threshold response (pSTR) while structural and biochemical alterations were evaluated through H&E staining, western blotting and immunohistochemical analysis of the retinal tissues.

**Results:** A greater loss of pSTR amplitudes was observed in the WT mice compared to Cav-1^-/-^ retinas in both the models. Silencing of Shp2 phosphatase imparted protection against inner retinal function loss in chronic glaucoma model in WT mice. The functional rescue also translated to structural preservation of ganglion cell layer in the chronic glaucoma condition in WT mice which was not evident in Cav-1^-/-^ mice retinas.

**Conclusions:** This study indicates that protective effects of Shp2 ablation under chronic experimental glaucoma conditions are dependent on Cav-1 in the retina, suggesting *in vivo* interactions between the two proteins.

## Introduction

Glaucoma is the second leading cause of irreversible vision loss, characterised by optic neuropathy and progressive damage to the retinal ganglion cells (RGCs) 1. The disease is estimated to contribute to approximately 8.4% cases of blindness amongst 50 years and older population in 2015 2, 3. It is also expected to affect around 111.8 million people worldwide by 2040 4. Enhanced intraocular pressure (IOP) is one of the noticeable features of the disease, and the chronic injury could be mediated by combination of one or more of the risk factors including ischemia, oxidative stress, age and neurotrophic obstruction due to impaired axoplasmic flow 5-7. Accordingly, identifying a single principal mechanism underlying glaucoma pathology or adversely affecting the RGCs has remained elusive 8-10. Despite therapeutic lowering of IOP, many glaucoma cases continue to progress with evidence of visual deterioration and optic nerve damage implicating IOP-independent mechanisms in the pathological process 11, 12.

Cumulative evidence suggests the pivotal role of neurotrophins and in particular brain-derived neurotropic factor (BDNF), in supporting the health and survival of RGCs, in various models of optic nerve injury 13-16. Neurotrophin administration has consequently been shown to protect the RGCs in experimental models of glaucoma 5, 17, 18. BDNF administration has been shown to delay RGC apoptosis and extend neuronal survival under various stress conditions 19-21. BDNF primarily mediates its neuroprotective effects through its high affinity receptor tropomyosin receptor kinase B (TrkB) which is well expressed in the retinal ganglion cells and lamina cribrosa 22-24. In addition BDNF has also been shown to be retrogradely transported to the RGCs from the brain through axoplasmic flow 25. Activation of TrkB receptor in the ganglion cells leads to activation of MAPK and PI3K signalling which are involved in neuronal growth, survival and neuritic growth 14, 15, 26. SH2 domain-containing tyrosine phosphatase-2 (Shp2; PTPN11) is implicated in negative regulation of TrkB through inhibition of BDNF induced TrkB phosphorylation in neuronal cells 27-29. Shp2 is ubiquitously expressed in the brain and retina and plays important role in cell proliferation, differentiation and migration 30-33. Previous studies have shown that conditional ablation of phosphatase resulted in developmental problems in the brain and eye 29, 34, 35. Shp2 has also been implicated in regulating cytoskeleton arrangement 36 and neurotrophin networks including BDNF/TrkB signalling 28, 29.

In the retina, Shp2 is well expressed in the ganglion cell layer (GCL) and inner nuclear layer (INL) suggesting its roles in maintaining normal retinal physiology 37, 38. Cai et al. (2011), described Shp2 to play a role in retinal progenitor cell fate and emphasised ERK survival mechanism to play a role in neural retina, although the phosphatase expression did not seem to be decisive in mediating retinal differentiation in later stages of development 35. More recently, we demonstrated that Shp2 negatively regulated the TrkB receptor phosphorylation and affected the receptor activity under glaucomatous conditions. Pharmacological inhibition of Shp2 and AAV2 mediated Shp2 silencing were both effective in restoring the TrkB activity 29, 38, 39. The Shp2 co-immunoprecipitated with TrkB and this interaction was observed to be mediated through the adapter protein Caveolin-1 (Cav-1) in the cultured RGCs 29. Cav is a key constituent of caveolae 40 and comprises three isoforms: Cav-1, Cav-2 and Cav-3 41. Cav-1 and 2 are ubiquitously expressed in non-muscle cells while Cav-3 is predominantly a muscle-specific isoform 42,43. Cav-1 shows a high immunoreactivity in the retinal GCL 44,45 and the gene locus was identified to be associated with glaucoma in genome-wide association studies (GWAS) 46-49. This study aimed to investigate the pathophysiological significance of Cav-1-Shp-2 interactions and their possible role in mediating RGC damage in animal models of glaucoma.

Further, Shp2 and Cav-1 proteins have both been implicated in regulating integrin signalling in neuronal cells 50-52. Integrin receptor expression in RGCs plays crucial roles in cellular survival and regeneration 53-56. Herein, we have investigated the effects of Shp2 modulation on BDNF/TrkB and integrin signalling using AAV mediated Shp2 modulation in the RGCs using CAG promoter. The *in vivo* interactions of Shp2 and caveolin-1 in RGCs are examined by specific modulation of Shp2 in the Cav-1^-/-^ mice. The study provided compelling evidence that Shp2 silencing, imparts protection to the retina against functional and structural deficits in chronic glaucoma model. The Cav-1^-/-^ mice studies highlight that Shp2 effects in the inner retina are mediated through Cav-1 and provide an evidence of physiological crosstalk between the two proteins *in vivo*.

## Methods

### Animals

Wild-type and Cav-1^-/-^ (KO) mice with C57BL/6 background (4-6 weeks) were used in this study. Animals were bred and housed under standard 12-h light and dark cycle condition with free access to food and water. All the animal experiments in this study were approved by Macquarie University Animal Ethics Committee (AEC) in Australia. The procedures were performed in accordance with the Australian Code of Practice for the Care and Use of Animals for Scientific Purposes and the guidelines of the ARVO Statement for the use of animals in Ophthalmic and Vision Research. For procedures including electroretinogram recordings, intravitreal administration of AAV construct and stimulating acute model of ocular hypertension, animals were anaesthetized using intraperitoneal injection of ketamine (50 mg/kg) and medetomidine (0.5 mg/kg) while isoflurane inhalation anaesthesia was used for IOP measurements and microbead administration (chronic model).

### AAV vector construct design

Pre-validated recombinant adeno-associated-virus 2 (AAV2) vectors were designed for either Shp2 overexpression or Shp2 knockdown (Vector Biolabs, USA). Briefly, for gene overexpression, murine Shp2 cDNA was placed under the transcriptional regulation of the CAG2 promoter. This synthetic promoter is composed of cytomegalovirus (CMV) enhancer fused to chicken β-actin transcription initiation site and rabbit β-globin intron. The promoter was introduced into the AAV2-eGFP vector (AAV2-CAG2-EGFP-T2A-mShp2 or briefly referred to AAV2-Shp2) (BC057398, isoform-a). Enhanced green fluorescent protein (eGFP) and Shp2 gene sequences were driven by CAG2 hybrid promoter including 2A linker. eGFP control vector was also expressed under the control CAG promoter flanked by AAV terminal repeats (briefly referred to AAV-GFP) and used as a control for Shp2 overexpression. For Shp2 silencing, mouse Shp2 shRNAmir was introduced in construct to achieve the knock down the Shp2 expression (AAV2-CAG-EGFP-mShp2-shRNAmir or AAV2-Shp2KD). The recombinant AAV2-Shp2KD construct targeted the Shp2 nucleotide coding sequence (NM_001109992.1) at 1296-(5'-CCTGATGAGTATGCGCTCAAA-3') site. Vectors encoding scrambled nucleotides sequence as reported previously were used as control (5'-GTCTCCACGCGCAGTACATTT-3') and indicated as (AAV-CAG-GFP-shRNA) 38, 39.

### Intravitreal AAV injections

Mice were anesthetized, and eyes dilated using 1% tropicamide as previously reported 16. AAV2 construct solution (final concentration, 1.8 × 10^12^ GC/mL) was carefully administered (2 µl) through the sclera at a 45° into the vitreous towards the *ora serrata* and posterior to the temporal limbus avoiding contact with lens. Injection was performed using 33G needle connected to 5 µl Hamilton syringe (Hamilton AG) guided by a surgical microscope (Carl Zeiss) to facilitate accurate focusing and a period of 30 seconds was allowed before removing the needle to permit diffusion of the virus in order to prevent leakage from the injection track. Animals were monitored and sacrificed at specified time points as indicated.

### Microbead induced chronic ocular hypertension model

A progressive elevation in IOP was achieved through the weekly injections of polystyrene microbeads (FluoSpheres polystyrene microspheres, 10 µm) in the anterior chamber for eight weeks as previously described 29. Briefly, mice were anesthetized through inhalation of isoflurane (2%), placed on a heating pad (36-37°C). Pupil dilation was performed by 1% tropicamide and topical anaesthesia applied as 0.5% alcaine eye drops respectively. Polystyrene microbeads (3.6×10^6^ microbeads/mL, 2-3 μl) were injected using 33-gauge needle (TSK Laboratory, Japan) connected to a 10 µl Hamilton syringe under the guidance of operating microscope (Carl Zeiss,) to facilitate accurate focus of the syringe with care taken to avoid touching iris or lens. IOP was bilaterally measured weekly by an iCare TonoLab (Icare, Finland) and the average from four consecutive reading was taken as the IOP at that specific time point.

### Acute intraocular pressure elevation model

Mice were also subjected to acute elevation of IOP as previously described 57-59. Briefly, mice were anesthetized and after applying mydriasis and corneal anaesthesia using 1.0% tropicamide and 1% proparacaine respectively, the anterior chamber of the eye was cannulated with 33G needle which was linked to an external saline reservoir elevated to induce a pressure of 60 mmHg for 1 h. In the control groups, eye was cannulated and maintained under normal pressure. Animals were allowed to recover and subsequently euthanised after two weeks and the tissues harvested.

### Electroretinogram (ERG) recordings

ERG recordings were acquired using OcuScience machine (OcuScience, USA) as previously described 60. Mice were dark-adapted overnight (> 12 hrs) and recordings carried out at the baseline and eight weeks following microbead model induction and two weeks after subjecting animals to acute model of ocular hypertension. The recordings were carried out under dim red-light illumination, after anaesthesia and pupil dilation using 1% tropicamide. Briefly, mice were positioned on the heating pad and stainless-steel reference and ground electrodes (OcuScience) positioned on the tail and subcutaneously over the forehead. Two silver embedded electrodes were positioned on the cornea and care taken to maintain the connection of the cornea and eye recordings electrodes using methylcellulose. Positive scotopic threshold (pSTR) recordings were obtained using a series of dim stimulation (-3.4 log cd·s/m^2^) delivered 30 times (frequency 0.5 Hz) and peak amplitudes were measured at 120 ms after flash stimulus 61, 62.

### Histology and staining of retinal sections

Animals were sacrificed at specific time points and transcardially perfused with 4% paraformaldehyde (PFA). Eyeballs were then harvested for morphometric evaluations, fixed in 4% PFA for 2 hrs, incubated overnight in 70% ethanol at 4°C and ultimately processed by automatic tissue processor (ASP200S, Leica). Eyes were subsequently embedded in paraffin ensuring their similar orientation using tissue marking dye and sections were made with the thickness of 5-7 µm 63. Sections were subjected to hematoxylin and eosin (H&E) staining as previously described 16, 63 and images were acquired using Zeiss microscope (Zeiss). For morphological analysis GCL cell density was measured over 500 µm (100-600 µm) from the edge of the optic disk.

For cryosections and immunofluorescence studies, eyes were fixed in 4% freshly prepared PFA for 2 hrs, washed with PBS incubated overnight in 4% sucrose, embedded in OCT cryo- media and stored at -80°C prior to immunohistochemical staining. Samples were then cryosectioned (5 µm) and subsequently blocked and permeabilised for 60 min in blocking buffer, washed and slides incubated overnight with primary antibodies prepared in antibody dilution buffer (1x PBS, 1% BSA, 0.3% Triton X-100) at 4°C.

Following antibodies were used as per given dilutions: anti-pCav-1 Y^14^ (abcam131235; 1:200), p-TrkB (p Y^515^) (abcam131483; 1:1000), anti-TrkB (sc-20542; 1:100), BDNF (sc-546; 1:250), anti-GFP (ab290; 1:250), anti-Shp2 (abcam131541; 1:250), anti-p-Shp2(Y^542^) (abcam62322; 1:250), anti-Brn3a (MAB1585; 1:300), anti-β1 Integrin (ab95623; 1:50), anti p-FAK(Y^397^) (ab81298; Cell signalling 9661; 1:100), anti-Cleaved Caspase-3 (Asp^175^) (1:100). Slides were subsequently subjected to three sequential washes with 1x PBS and incubated with the secondary antibodies as indicated (Cy3, Alexa Fluor 488 or Alexa Fluor 647) (1:400 dilution) for 2 hrs at room temperature. The coverslips were mounted on sections using anti-fade mounting medium with Prolong DAPI (Life Technologies) and imaged.

**Western blot analysis**

The optic nerve head (ONH) regions were surgically excised, and tissues lysed in the lysis buffer (1% Triton X-100, 2 mM EDTA, 50 mM NaF, 50 mM Tris pH8, 0.5% Sodium deoxycholate, 0.1% SDS, 150mM NaCl) containing PhosSTOP (Sig-Aldrich) and protease inhibitor (Merck). Protein concentration was determined using bicinchoninic acid (BCA) kit (Pierce, Rockford, IL, USA) and an identical amount of protein (25 µg) was resolved by on 10% SDS-PAGE and transferred to polyvinylidene difluoride membrane (PVDF). The membrane was blocked in Tris-buffered saline (TTBS) (20 mM Tris-HCl [pH 7.4], 100 mM NaCl and 0.1% Tween 20) including 5% skim milk for 1 h at room temperature and incubated overnight with primary antibodies at 4°C. The antibodies used and dilutions were: TrkB (1:1000), BDNF (1:500), p-Cav-1 (pY^14^) (1:500), p-TrkB (pY^515^) (1:500), Shp2 (1:1000) and p-Shp2 (p Y^542^) (1:500), GAPDH (Sigma-Aldrich G8795; 1:10000), anti-GFP (1:1000), anti-β1 Integrin (1:250) and anti p-FAK Y^397^ (1:250). The next day blots were subjected to three subsequent TBST washes followed by incubating with horseradish peroxidase (HRP)-labelled secondary antibodies (R&D system) at room temperature for one hour. Signals were visualised using an automated luminescent image analyser (ImageQuant LAS 4000) and densitometric analysis of band intensities carried out using ImageJ software (ImageJ, NIH, USA) 64,65. Values were normalized by re-probing the membranes with actin (internal control), and relative density of the peaks plotted 66.

### Apoptosis assay (TUNEL staining)

To detect apoptotic cells in GCL, we assessed both the activation of caspase 3 (casp3) using immunostaining against cleaved Casp3 (c-casp3) as well as TUNEL assay. Paraffin-embedded retinal sections were stained with TdT-mediated dUTP nick end labelling (TUNEL) assay technique using DeadEnd Fluorometric TUNEL System kit (Promega) according to the manufacturer's instructions. Briefly, retinal sections were deparaffinized using xylene and rehydrated through a series of decreasing concentrations of ethanol. Sections were subsequently subjected to permeabilisation using Proteinase K (20 µg/ml) and incubated with TUNEL reaction mixture (containing equilibration buffer, Nucleotide mix and rTdT enzyme) for 60 min at room temperature in a humidified chamber kept in dark. A positive control slide was similarly processed while also treated with DNase I prior to incubation with reaction mixture. The action was stopped by 2x saline-sodium citrate (SSC) buffer, and sections counterstained and mounted with antifade DAPI. Sections were examined using fluorescence microscope (Carl Zeiss) at 520 nm. TUNEL positive cells in GCL were counted over 500 µm (100-600 µm from the edge of the optic disk) and averaged in 4 successive sessions from each group and compared with the controls (n = 6 in each group).

### Statistical analysis

Statistical analysis for all data including ERG/pSTR amplitudes, GCL density, western blot, TUNEL assay and histology was performed using Graphpad Prism (v 8.2.1) (GraphPad Software, San Diego, CA). In all represented data, values and error bars are expressed as mean ± SD from given n sizes. Statistical analysis performed using either Student's t-test for unpaired data or one-way ANOVA followed by Bonferroni's post-hoc multiple comparisons test. The significance was set at p < 0.05.

## Results

### Shp2 regulatory effects on the inner retina are mediated through Cav-1

**Figure 1:**
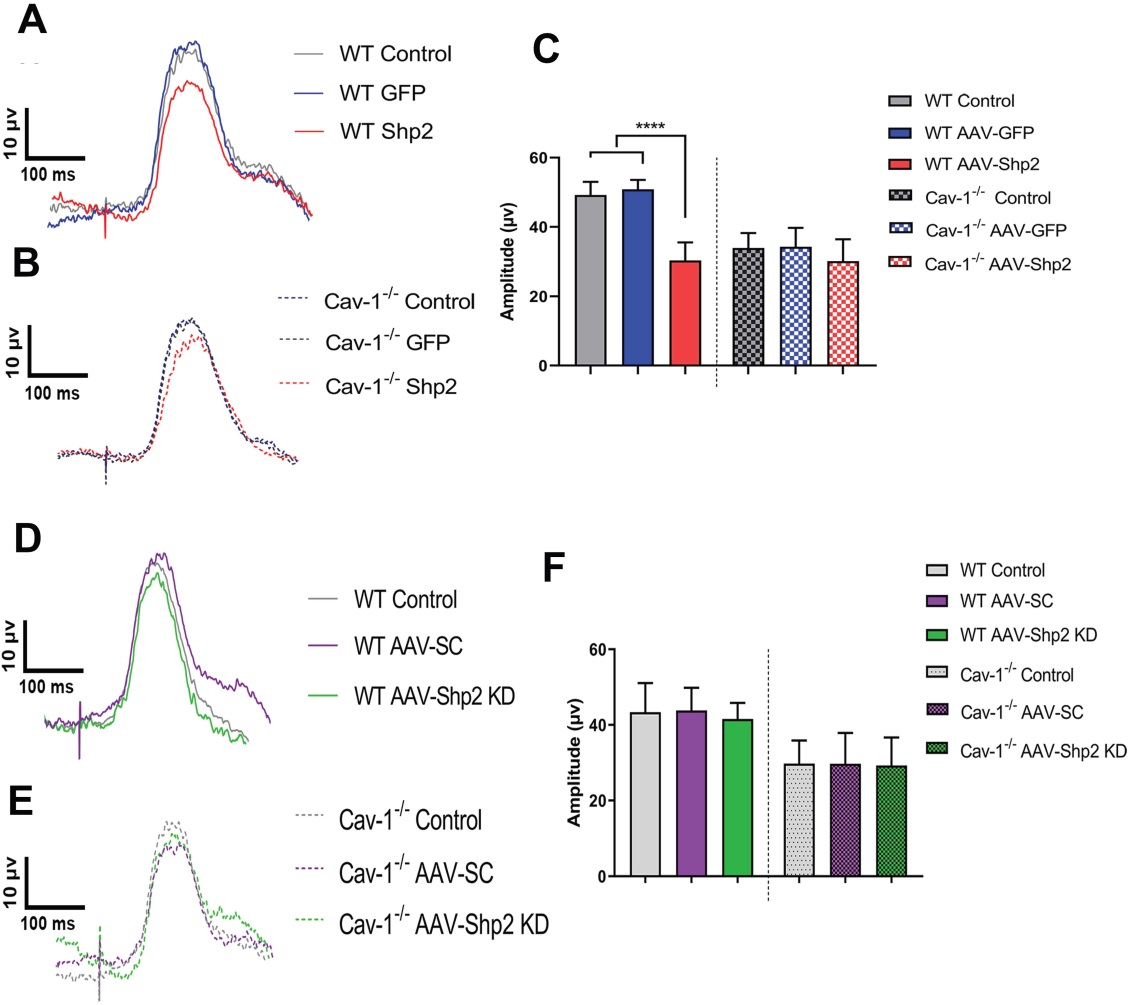
** Cav 1^-/-^ mice retinas were relatively protected against the functional loss induced upon Shp2 upregulation**. A, B) Average trace of pSTR signal from AAV-Shp2 treated WT and Cav-1^-/-^ mice and corresponding controls 2 months following treatment. (C) Quantification indicates that AAV-Shp2 injected WT mice had significantly lower pSTR amplitudes in comparison to AAV-GFP counterparts. (****p < 0.0001, WT; Cav-1^-/-^ mice: p = 0.17; n = 16) (C) Average traces of pSTR amplitudes in WT and Cav-1 KO retinas. (D-F) No significant changes were observed in pSTR amplitudes in either WT or Cav-1 KO groups retinas following Shp2 downregulation under normal healthy conditions.

This study demonstrates that Shp2 effects on the retina are mediated through an adapter protein Cav-1. We sought to investigate the potential *in vivo* interactions between Shp2 and Cav-1 proteins by modulating Shp2 expression in the inner retina using AAV-Shp2 and AAV-shRNAmir constructs in WT and Cav-1^-/-^ mice (Figure S1A, B). Electrophysiological responses in the retina were measured two months following AAV2 gene therapy. A significant decline in pSTR amplitudes was observed 2 months following Shp2 upregulation in AAV-Shp2 injected WT animals (WT AAV-GFP (49.23 ± 3.78) compared to their AAV-Shp2 counterparts (30.33 ± 5.21 µV) (****p < 0.0001, one-way ANOVA, Bonferroni post-hoc test; n = 8) while the AAV-Shp2 treated Cav-1^-/-^ mice, only demonstrated a slight decrease in inner retinal function which was not statistically significant (34.30 ± 5.40 in Cav-1^-/-^ control *vs* 30.11 ± 6.33 µV in AAV-Shp2 Cav-1^-/-^) (Figure 1A-C). We have previously reported that Cav-1^-/-^ mice exhibit lower pSTR amplitudes under normal conditions compared to the WT animals 67. Shp2 silencing in contrast did not lead to any significant differences in the retinal function of either WT or Cav-1^-/-^ group (Figure 1D-F).

**Figure 2:**
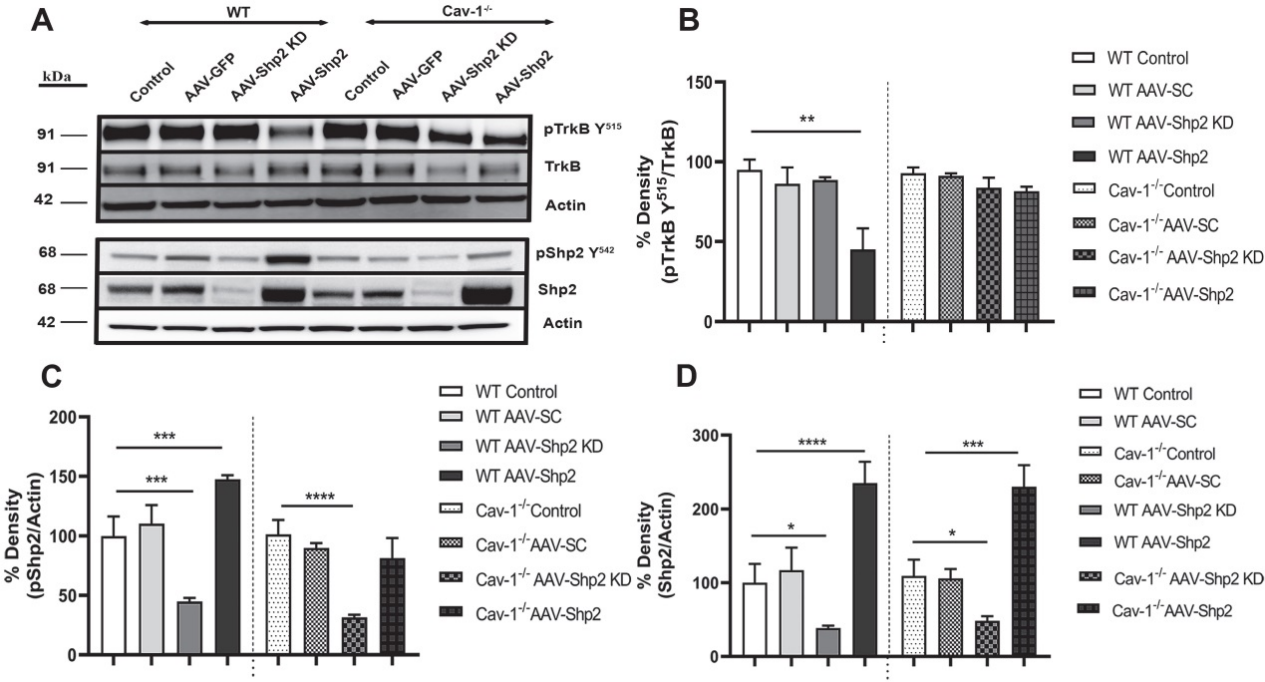
** Shp2 upregulation resulted in retinal TrkB receptor dephosphorylation** (A) Western blot indicating pTrkB Y^515^/TrkB, pShp2 and Shp2 expression under control and AAV-Shp2 modulated conditions. (B). Multiple comparisons using Bonferroni post-hoc test showed that there was a significant decrease in pTrkB (**p < 0.01) which was concurrent with higher pShp2 levels (***p < 0.001) (C) in WT retinas following Shp2 upregulation. Cav-1^-/-^ GCL did not show significant decline in pTrkB and total TrkB levels upon Shp2 downregulation. (D) Significant reduction in Shp2 expression was observed upon using AAV-mShp2 shRNA in both WT and Cav-1 ^-/-^ mice (*p < 0.05, one-way ANOVA; n = 24) while increased expression levels were observed following AAV-Shp2 administration (****p < 0.0001, WT; ***p < 0.001, Cav-1 ^-/-^; one-way ANOVA; n = 24).

Shp2 is involved in negative regulation of TrkB signalling in the stressed RGCs 29, 38 , therefore we analysed changes in TrkB and pTrkB Y^515^ as well as Shp2 and pShp2Y^542^ expression in the WT and Cav-1^-/-^ mice (Figure 2 A-D). We observed 2.3 ± 0.28 to 2.1 ± 0.26-fold increase in Shp2 expression in AAV-Shp2 administered WT and Cav-1^-/-^ retinal samples respectively (****p < 0.0001, WT; ***p < 0.001, Cav-1^-/-^ one-way ANOVA, Bonferroni post-hoc test; n = 6). The expression reduced by an average of 2.59 ± 0.23 and 2.25 ± 0.31-fold in AAV2-Shp2 shRNAmir injected eyes (*p < 0.05 in both groups) when compared to control samples (Figure 2 A, D). Immunofluorescence analysis of retinal sections further established a remarkable increase of Shp2 expression in GCL of the AAV2-Shp2 transduced eyes, while a significantly reduced expression of Shp2 was observed in the AAV2-Shp2-shRNA administered retinas (Additional file 1: Figure S1 C-F). Brn3a staining was used as RGC marker to localize Shp2 and GFP expression in the GCL (Additional file 1: Figure S2).

Subsequent to Shp2 upregulation, pTrkB Y^515^ immunoreactivity was reduced by approximately 44 ± 15.4 % as compared to the eGFP alone transduced group in WT mice. A decline of 25 ± 8.5% pTrkB reactivity was observed in Cav-1 null retinas. These results suggest that shp2 modulatory effects on TrkB are facilitated by Cav-1. A 1.5-fold ± 0.03 increase in pShp2 Y^242^ which is activated form of the phosphatase was also observed (Figure 2A, C). These findings are in agreement with previous studies which have demonstrated that Shp2 increase hindered BDNF-induced TrkB phosphorylation through its negative modulation of TrkB signalling 28. Cav-1 deficient retinas however, showed lesser degree of alterations in either TrkB or Shp2 phosphorylation (Figure 2 A-C) in GCL, further supporting the argument that Shp2 effects on TrkB are mediated through Cav-1 in the retina. No significant changes in the expression of total TrkB receptor expression were observed (Figure 2 A, B).

**Figure 3:**
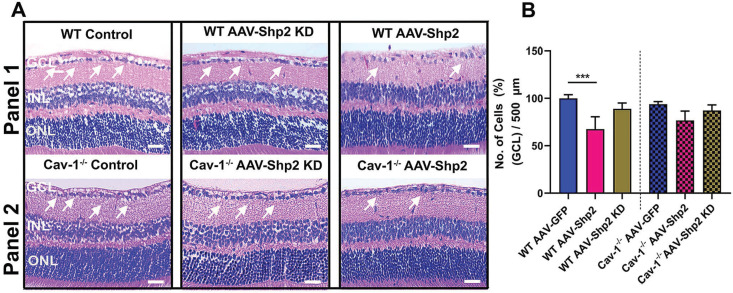
** Histochemical evaluation of WT and Cav-1^-/-^ retinas two months following Shp2 modulation** (A) H&E staining of paraffin-embedded retinal sections indicating different retinal layers including GCL in WT and Cav-1^-/-^ groups (Scale bar = 50 µm). (B) Quantification revealed significantly reduced GCL density in AAV-Shp2 treated WT mice compared to controls (***P < 0.001, one-way ANOVA; n = 4 in each group) while no significant changes in GCL density were identified in case of AAV-Shp2 injected Cav-1 KO mice. (A; panel 2). Further, no significant changes were observed upon Shp2 KD in the WT or Cav-1^-/-^ mice.

We also aimed to examine whether Shp2 expression modulation and resultant decline in inner retinal function was related to potential changes in the retinal laminar structure. Morphological assessment using H&E staining indicated reduced GCL density two months post AAV-mediated Shp2 upregulation (***p < 0.001; one-way ANOVA; n = 8) (Figure 3A, B). WT mice retinas lost a significantly higher proportion of cells in the GCL (1.4-Fold higher) in comparison to Cav-1^-/-^ counterparts (WT; ***p < 0.001; one-way ANOVA; Benferroni post-hoc test n = 4). In contrast, no significant structural alterations in GCL were observed to be associated with Shp2 silencing (Figure 3A, B).

### Shp2 silencing protected retina against chronic glaucoma injury in a caveolin dependent manner

**Figure 4:**
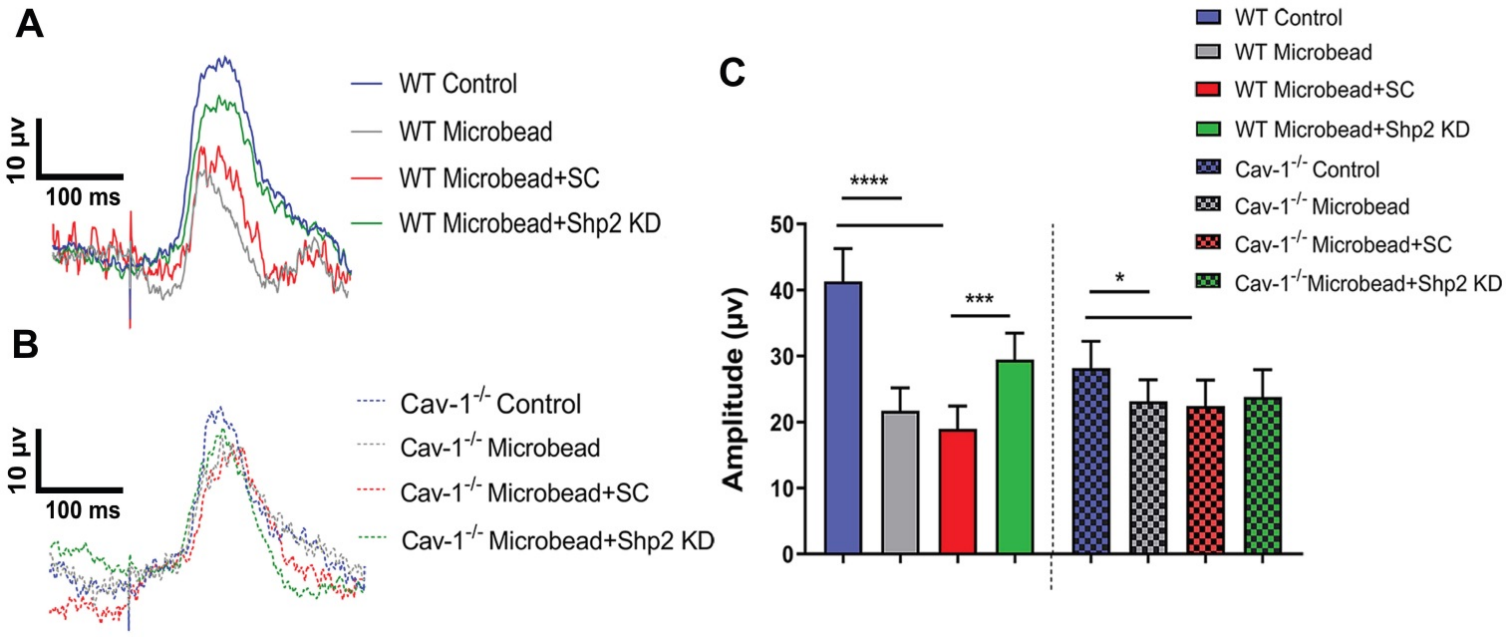
** Silencing of Shp2 protected the inner retinal function in WT but not Cav-1 KO retinas**. (A, B) pSTR responses in WT and Cav-1^-/-^ retinas in control and experimental groups treated with AAV-Shp2 scramble (sc) and AAV-Shp2 KD under chronic glaucoma conditions. (C) A significant decline in pSTR observed in AAV-Sc treated WT mice exposed to chronic elevation of IOP (****p < 0.0001; one-way ANOVA; Bonferroni post-hoc test; n = 16) as against the AAV-Shp2 KD retinas that were rescued under similar conditions (***p < 0.001; one-way ANOVA; Bonferroni post-hoc test; n = 16). AAV-Sc treated Cav-1^-/-^ retinas demonstrated reduced loss of pSTR amplitudes compare to WT (*p < 0.05, Cav-1^-/-^ and Cav-1^-/-^ microbead) and Shp2 knockdown did not induce a protection of the retinal function.

Previous studies suggest that Shp2 interacts with Cav-1 adaptor protein in both neuronal and non-neuronal cells 29, 68. Given that upregulation of Shp2 expression resulted in impaired inner retinal function in WT but the effects were less significant in Cav-1 ablated retinas; we examined whether Shp2 silencing could protect inner retinal function in glaucoma and tested this premise in two different models of experimental glaucoma. IOP was elevated in response to microbead injection and it ranged from 24.10 ± 5.17 mmHg and 26.12 ± 6.40 mmHg in WT and Cav-1 KO compared to the baseline IOP of 10.78 ± 0.36 mmHg and 12 ± 1.02 mmHg for control mice respectively (Additional file 1: Figure S3). Electrophysiology recordings illustrated a significant loss of pSTR amplitudes in both WT and Cav-1^-/-^ groups in response to IOP elevation. A greater decline was observed in WT compared to the Cav-1^-/-^ animals (****p < 0.0001, WT and WT microbead; *p < 0.05, Cav-1^-/-^ and Cav-1^-/-^ microbead) (Figure 4A-C). Further analysis established a significant protection of the inner retinal function (pSTR) in WT mice following silencing of Shp2 (18.96 ± 3.47 µV in WT microbead; 29.46 ± 4.03 µV in WT microbead+Shp2 KD) (***p < 0.001; one-way ANOVA; Bonferroni post-hoc test; n = 16). Shp2 knockdown in Cav-1^-/-^ mice did not reveal a significant protection against the inner retinal functional deterioration (p = 0.5; n = 16) (Figure 4B, C). This implied that Shp2 effects are mediated at least partly through Cav-1 and that silencing phosphatase in the absence of Cav-1 was not effective in rescuing pSTR impairment.

**Figure 5:**
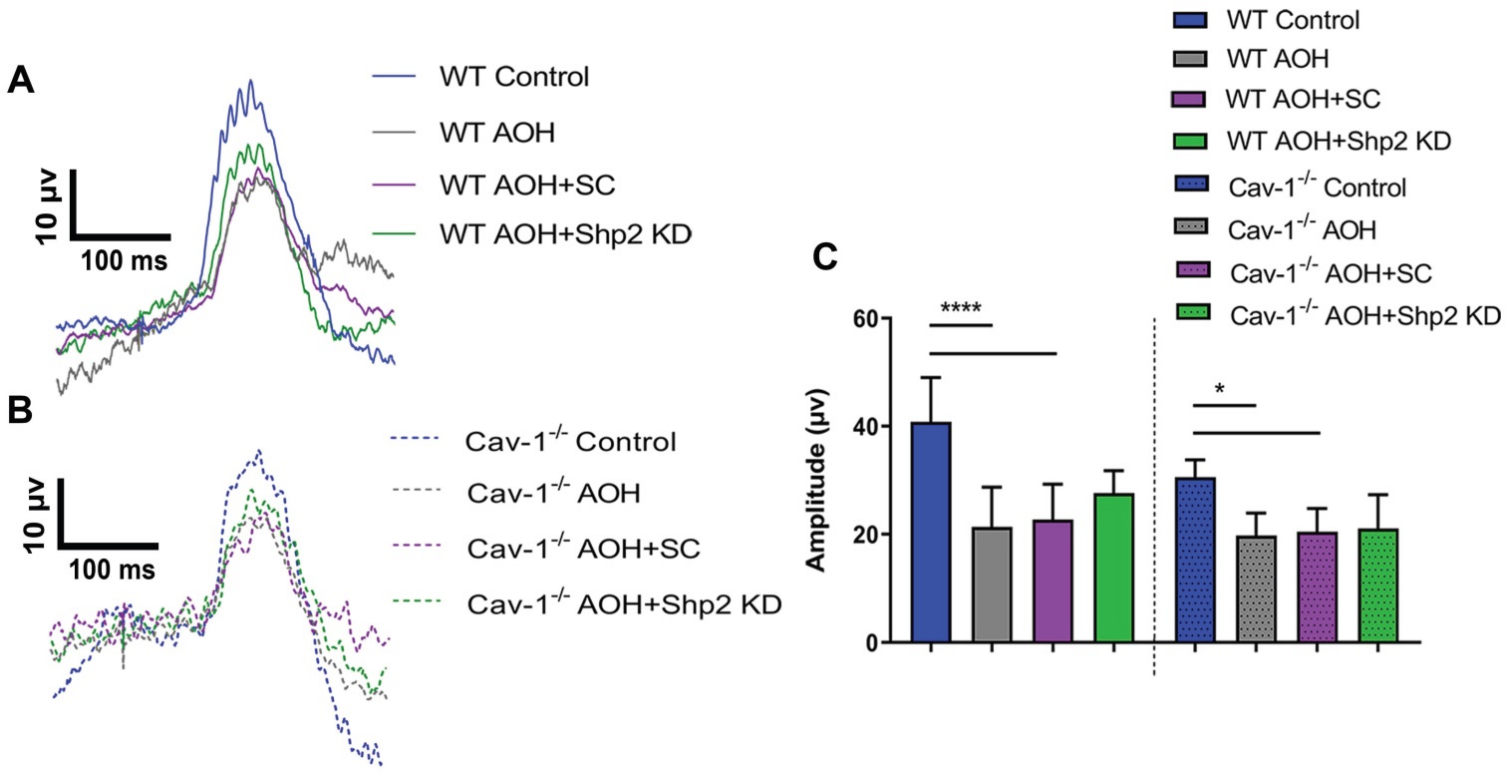
** AOH induced inner retinal impairment was not rescued by Shp2 silencing.** (A, B) pSTR traces in WT and Cav-1^-/-^ eyes exposed to acute IOP increase and Shp2 downregulation using AAV-Shp2 KD. (C) Quantification of pSTR amplitudes revealed statistically significant decrease in inner retinal function in WT and Cav-1^-/-^ mice (****p < 0.0001, WT; *p < 0.05, Cav-1^-/-^; one-way ANOVA; Bonferroni post-hoc test; n = 16) which was not affected upon Shp2 KD.

The potential protective effects of Shp2 silencing on the inner retinal function were also investigated in acute ocular hypertension (AOH) model. In these animals, eyes were subjected to raised IOP of 60 mm Hg for 1 h as described previously and the animals monitored for 2 weeks 58, 59, 69. Exposure to acutely raised IOP led to loss of pSTR amplitudes in the retinas compared to the control eyes, although the relative decline was greater in WT compared to the Cav-1^-/-^ mice. Of note, in contrast to the chronic glaucoma model, Shp2 silencing in AOH only conferred marginal increase (17% ± 4.11) in pSTR amplitudes in WT retinas (40.83 ± 8.18 µV WT control; 22.71 ± 6.56 µV in WT AOH; 27.65 ± 4.11 µV in WT AOH+Shp2 KD) which was not statistically significant (Figure 5A, C). Similarly, Shp2 silencing did not translate into any significant protection in Cav-1^-/-^ animals (30.56 ± 3.19 µV Cav-1^-/-^ control; 20.48 ± 4.31 µV in Cav-1^-/-^ AOH; 21.09 ± 6.22 µV in Cav-1^-/-^ AOH+Shp2 KD) (Figure 5B, C). These results suggested that inner retinal injury induced by acute IOP elevation is independent of Shp2 modulatory effects.

**Figure 6:**
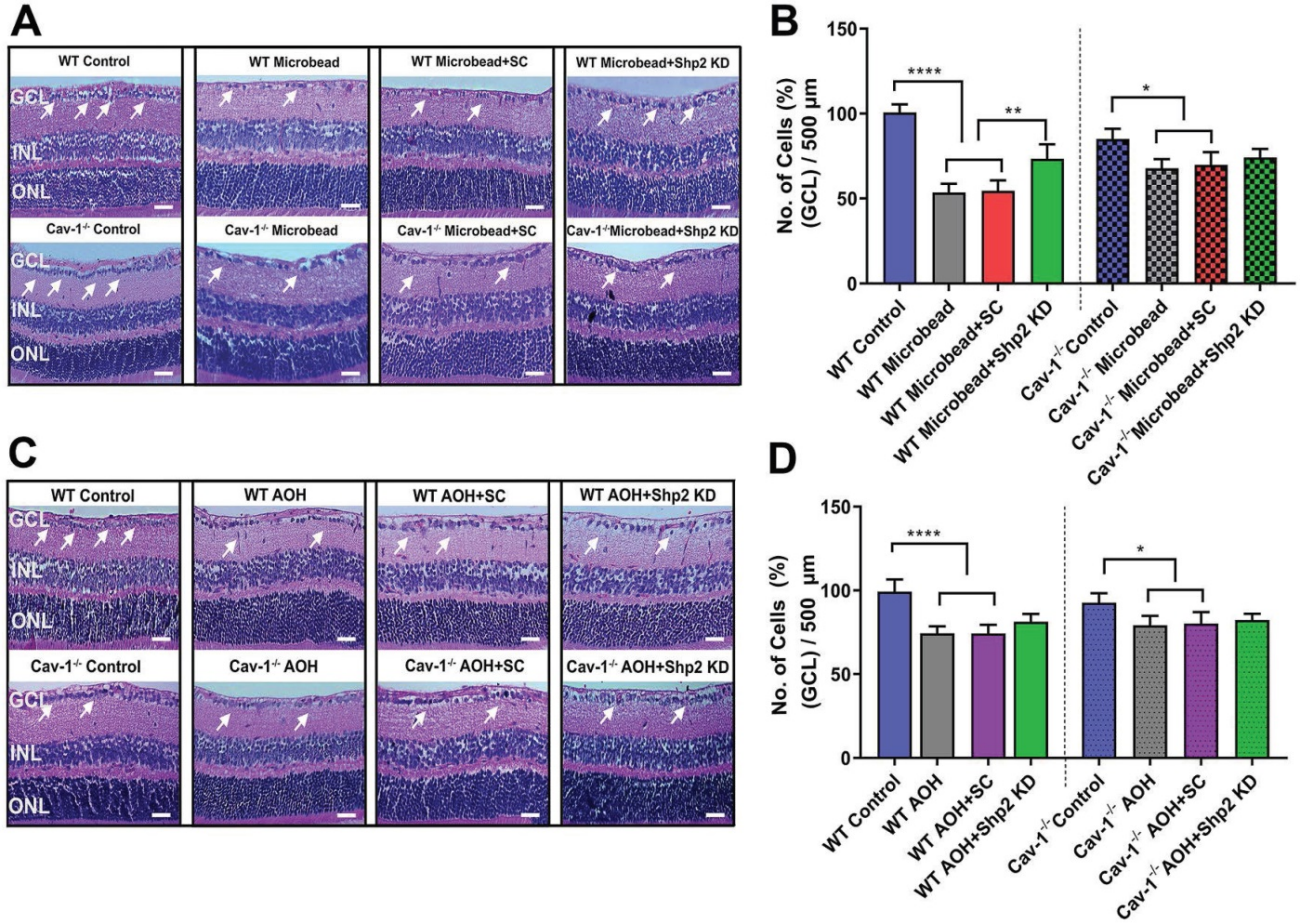
** Shp2 silencing protected inner retinal laminar structure in WT mice in chronic but not acute glaucoma model** (A, B) H and E staining of WT and Cav-1 null retinas two months after chronic high IOP and (C) 2 weeks following exposure to acute IOP (Scale bar = 50 µm). (B) Quantification of GCL density revealed that under chronically elevated IOP, a significant reduction occurred in WT (****P < 0.0001, one-way ANOVA, n = 8,) and Cav-1 ablated mice (*p < 0.05, one-way ANOVA, n = 8). Upon subjecting the RGCs to AAV-Shp2 KD, significant protection of GCL density was observed in WT (*p < 0.05, one-way ANOVA, n = 8) compared to Cav-1^-/-^ group. (D) H and E staining also indicated a significant loss in GCL density in acute high IOP (**P < 0.01, WT; *p < 0.05, Cav-1^-/-^. one-way ANOVA, n = 8) while downregulation of Shp2 resulted in no significant change in either WT or Cav-1^-/-^ mice.

We further examined whether functional rescue of Shp2 silencing in WT mice also translated into structural protection. Histological analysis using H&E staining of retinal sections demonstrated that while a significant decline in GCL density was identified in WT chronic glaucoma mice that were administered AAV scramble construct (****p < 0.0001; one-way ANOVA; Bonferroni post-hoc test; n = 8), GCL density was significantly preserved in the group subjected to Shp2 inhibition (**p < 0.01; one-way ANOVA; Bonferroni post-hoc test; n = 8) (Figure 6A, B). The Cav-1^-/-^ mice retinas exposed to chronically elevated IOP were relatively unaffected as a consequence of Shp2 silencing (*p < 0.05; one-way ANOVA; Bonferroni post-hoc test; n = 8). No protective effects were observed upon Shp2 inhibition in GCL loss in acute glaucoma model (Figure 6C, D).

**Figure 7:**
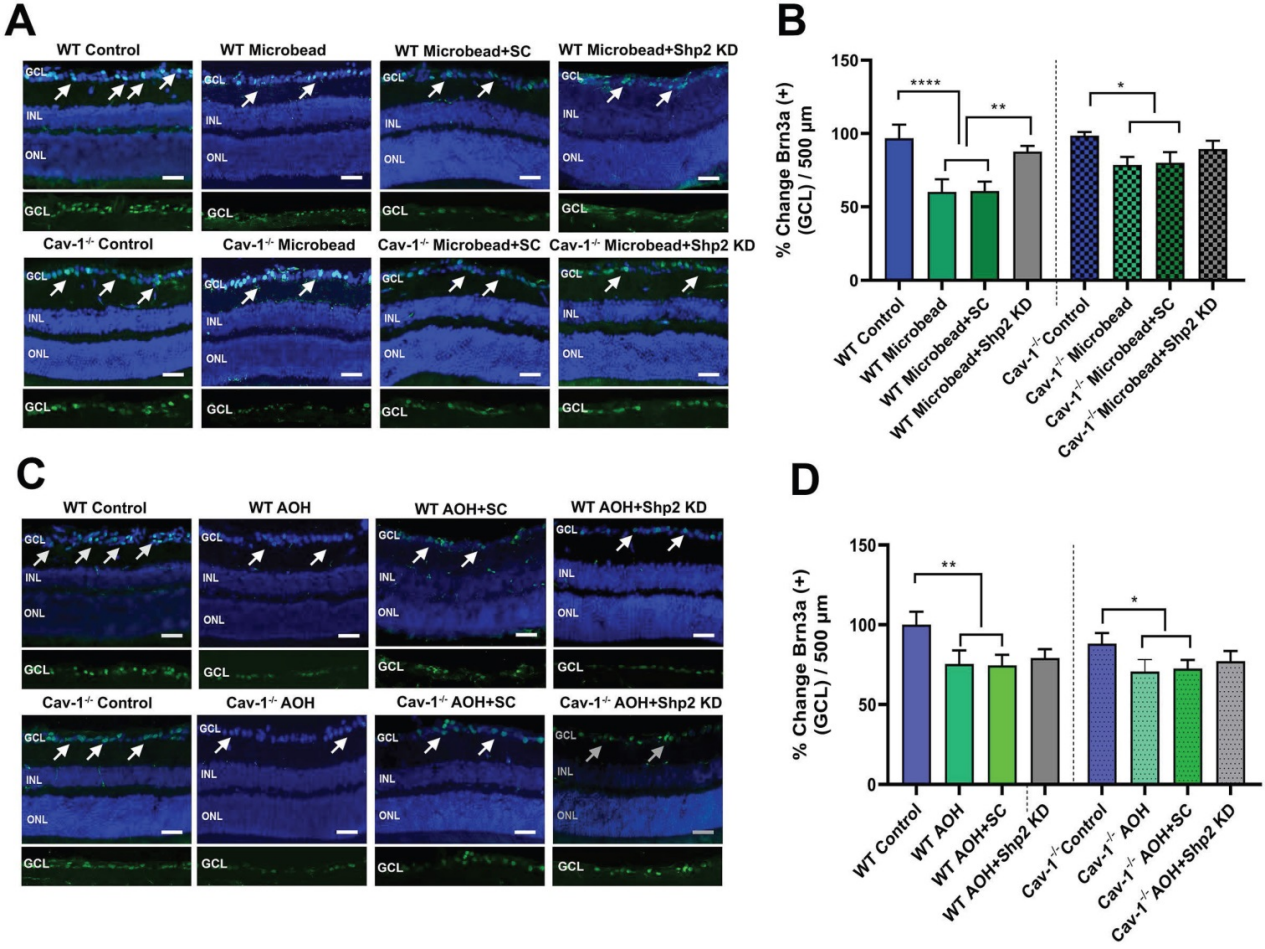
** Shp2 expression knockdown partially rescued IOP-induced loss of Brn3a positive cells in WT mice.** (A, C) Retinal sections immunostained with RGC marker Brn3a, two months following chronic and 2 weeks after acute glaucoma exposure (Scale bar = 50 µm). (B, D) WT retinas indicated a higher Brn3a^+^ loss following chronic high IOP exposure compared to the Cav-1 KO mice (****p < 0.0001, WT; *p < 0.05, Cav-1^-/-^. one-way ANOVA, n = 8). This loss was significantly diminished (**p < 0.01, one-way ANOVA, n = 8), in the WT group administered AAV-Shp2 KD construct compared to Cav-1^-/-^ animal retinas where no significant changes were observed under similar conditions.

This was followed by Brn3a immunostaining of the retinal sections 70,71 and the results (Figure 7) revealed that there was a significant decrease in Brn3a positive cells in WT mice in chronic glaucoma (****p < 0.0001; one-way ANOVA; n = 6) which was rescued under Shp2 KD conditions (**p < 0.01; one-way ANOVA; n = 6) (Figure 7A, B). Cav-1^-/-^ mice, however, did not demonstrate significant differences in Brn3a positive cell population in GCL after Shp2 suppression (*p < 0.05; one-way ANOVA; n = 6) (Figure 7A, B). In the acute model of IOP increase, Brn3a positive ganglion cells were reduced in both WT or Cav-1^-/-^ (Figure 7C, D) mice and suppression of Shp2 was not effective in imparting protection to the Brn3a positive cells in either WT or Cav-1^-/-^ mice (n = 6).

### Shp2 silencing rescued BDNF/TrkB signalling under chronically elevated IOP

**Figure 8:**
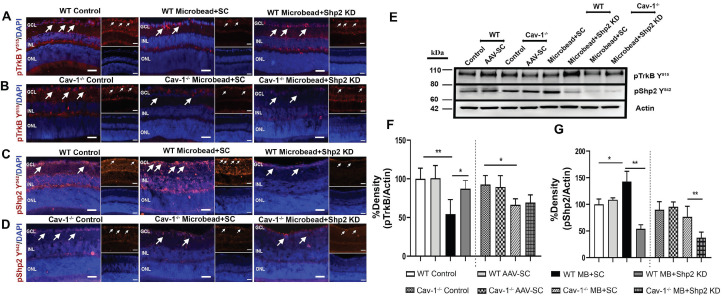
** Reduced levels of pTrkB in chronic glaucoma model were mitigated upon Shp2 silencing**. (A-D) Retinal sections immunostained with pTrkB (A, B and pShp2 (C, D) in WT and Cav-1^-/-^ retinas 2 months following chronic IOP elevation. DAPI was used for staining the nuclei. (Scale bar = 50 µm). (E) WB analysis of pTrkB and pShp2 levels in retinal ONH lysates. (F) Densitometric analysis indicated markedly reduced pTrkB immunoreactivity following chronic IOP elevation in WT and Cav-1^-/-^ GCL (**p < 0.01, WT, *p < 0.05, Cav-1^-/-^ one-way ANOVA, n = 6) which was concurrent with increased pShp2 (G) in WT group (*p < 0.05, one-way ANOVA, n = 6). Shp2 silencing protected against pTrkB decline (G) (*p < 0.05, one-way ANOVA, n = 6) in WT mice while no significant effects on pTrkB levels were observed in Cav-1 deficient retinas following similar levels of Shp2 KD.

Shp2 has been demonstrated to antagonise TrkB activity in the RGCs under stress conditions and this corresponded with hyperphosphorylation of Cav-1 protein 29, 38. Herein we demonstrate that TrkB phosphorylation was significantly reduced (1.9 ± 0.62 fold) in WT mice upon exposure to chronic IOP elevation as indicated by western blotting and immunofluorescence analysis (**p < 0.01, one-way ANOVA, n = 6) (Figure 8A, F). This decline in TrkB phosphorylation corresponded with an increase in pShp2 levels (Figure 8C, G) (*p < 0.05, one-way ANOVA, n = 6). In Shp2 knockdown subjected retinas, TrkB phosphorylation was observed to be significantly enhanced (*p < 0.05, one-way ANOVA, n = 6). In comparison, while we observed a significant reduction in pTrkB levels in Cav-1^-/-^ mice following exposure to chronically elevated IOP, (*p < 0.05, one-way ANOVA, n = 6), this loss was not rescued in response to Shp2 suppression. These results together indicate that Shp2 effects on TrkB phosphorylation are dependent on Cav-1 phosphorylation under chronic glaucomatous stress conditions. The expression of Cav protein did not reveal any changes under any of the conditions (Figure S4). This immunofluorescence staining of retinal sections also confirmed the absence of this protein in Cav-1 knockout retinas. Brn3a co-immunostaining revealed that pTrkB (Y^515^), pShp2 (Y^542^) and pCav-1 (Y^14^) were expressed in the RGCs (Figure S2).

**Figure 9:**
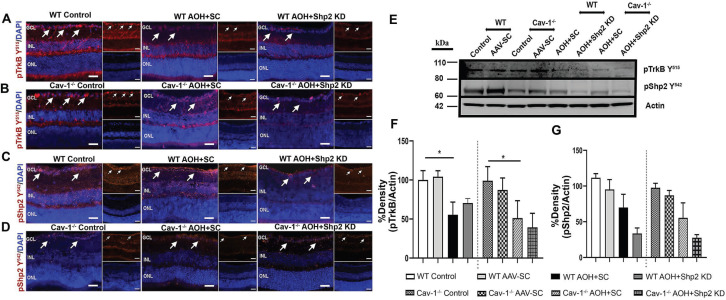
** Reduced pTrkB levels in acutely elevated IOP condition was independent of Shp2 silencing effects**. (A-D) IF images of retinal sections subjected to immunostaining with pTrkB (A, B) and pShp2 (C, D) in WT and Cav-1^-/-^ retinas two weeks following acute IOP exposure (Scale bar = 50 µm). DAPI used for staining the nuclei. (Scale bar = 50 µm). (E) Western blot indicating pTrkB and pShp2 levels in high IOP exposed retinal lysates. (F) TrkB receptor significantly lost its phosphorylation following acute IOP elevation in WT or Cav-1 null retinas (*p < 0.05, one-way ANOVA, n = 6). (G) pShp2 levels were significantly reduced in AOH model in Shp2 KD conditions.

The BDNF/TrkB signalling and pShp2 expression changes were also monitored in WT and Cav-1^-/-^ mice in the acute model of IOP increase subsequent to AAV-mediated Shp2 downregulation. Immunostaining performed with anti-pTrkB (Y^515^), anti-pShp2 (Y^542^) and anti-pCav-1 Y^14^ antibodies in IF and WB revealed that there was a significant decline in pTrkB levels in both WT and Cav-1 deficient retinas after acute IOP exposure (*p < 0.05, one-way ANOVA, n = 6), however no significant improvement in the status of TrkB phosphorylation was observed in either of these groups subsequent to Shp2 silencing (Figure 9 A, B and F). pShp2 Y^542^ levels were significantly increased in the retina in chronic glaucoma conditions in WT mice (Figure 8C, G). This increase was not evident in Cav-1^-/-^ mice in chronic model (Figure 8D, G) and was also not observed in either WT or Cav-1^-/-^ mice in acute ocular hypertension model (Figure 9 C, D and G). A comparable decrease in pShp2 levels were observed in response to Shp2 silencing in each of the WT and Cav-1^-/-^ models (Figure 8, 9).

### Shp2 knockdown reduced glaucoma induced apoptosis in a Cav-1 dependent manner

**Figure 10:**
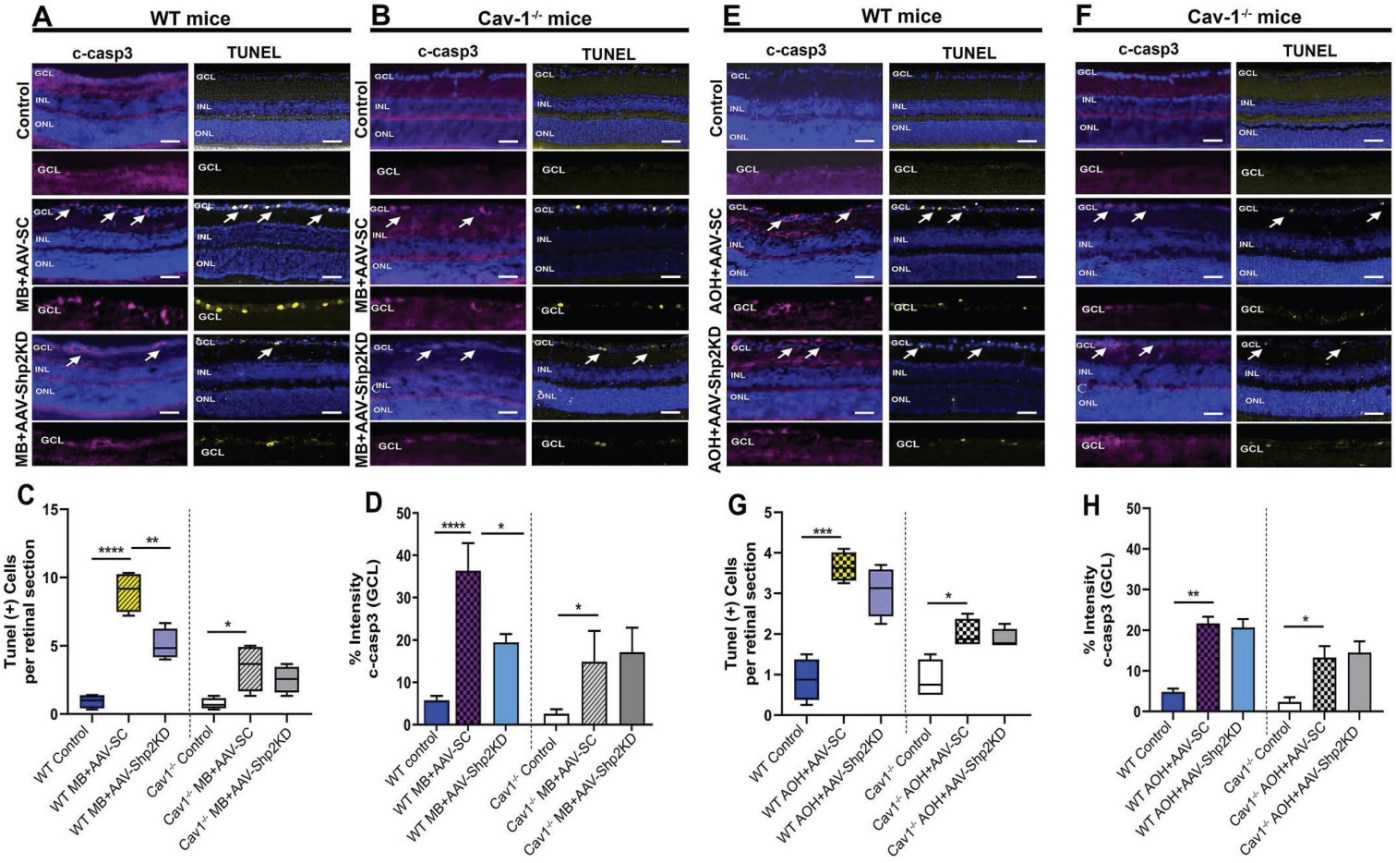
** Shp2 silencing alleviated apoptotic activation in GCL in a chronic model of glaucoma** (A, B) Retinal sections immunostained with both cleaved c-casp3 and TUNEL apoptotic markers (Scale bar = 50 µm). WT retinas depicted a higher TUNEL^+^ and c-casp3 immunoreactivity (A) following chronic IOP exposure when compared to their (B) Cav-1 KO counterparts. Significant decline in apoptotic staining and immunoreactivity of c-casp3 was observed subsequent to Shp2 silencing in WT retinas. (C) Quantification of TUNEL^+^ cells in GCL showed higher apoptotic activity in WT retinas after chronic elevation of IOP (****p < 0.0001, one-way ANOVA; Bonferroni post-hoc test; n = 8) which was considerably attenuated in the AAV-Shp2 KD subjected group (**p < 0.01, one-way ANOVA; Bonferroni post-hoc test; n = 8). Cav-1 ablated retinas illustrated apoptosis that remained relatively unaltered after shp2 loss (*p < 0.05, one-way ANOVA; Bonferroni post-hoc test; n = 8). (D) c-casp3 immunostaining established increased apoptosis in WT retinas subsequent to chronic IOP elevation (****p < 0.0001, one-way ANOVA; Bonferroni post-hoc test; n = 8). This increase in c-casp3 was mitigated in the WT group with Shp2 silencing effects (*p < 0.05, one-way ANOVA; Bonferroni post-hoc test; n = 8). Cav-1^-/-^ retinas exhibited lower c-casp3 immunoreactivity that remained unaltered in response to Shp2 silencing (*p < 0.05, one-way ANOVA; Bonferroni post-hoc test; n = 8). (E, F) TUNEL and c-casp 3 staining in WT and Cav-1 ablated retinas following acute model of experimental glaucoma (Scale bar = 50 µm). (G, H) Quantification analysis of TUNEL and c-casp3 staining established that both of these apoptotic cell markers were significantly enhanced in both WT (***p < 0.001, one-way ANOVA; Bonferroni post-hoc test; n = 8) and Cav-1^-/-^ retinas when subjected to acutely elevated IOP (*p < 0.05, one-way ANOVA; Bonferroni post-hoc test; n = 8). AAV mediated shp2 downregulation did not result in significant amelioration of apoptotic staining in either of these groups.

Several lines of evidence suggest that glaucoma is marked by increased apoptosis in the RGCs 16, 26. TUNEL apoptotic staining was performed on the retinal sections of WT and Cav-1^-/-^ KO animals in both chronic and acute models of elevated IOP. A significantly increased TUNEL positive cell population was observed in WT mice that were subjected to microbead induced IOP increase (****p < 0.0001; one-way ANOVA, Bonferroni post-hoc test; n = 8) (Figure 10A) compared to Cav-1 KO mice under similar conditions (*p < 0.05; one-way ANOVA; Bonferroni post-hoc test; n = 8) (Figure 10B). Statistical analysis further revealed that Shp2 suppression substantially alleviated apoptotic changes in WT mice (1.8 ± 0.3-fold; **p < 0.01; one-way ANOVA; Bonferroni post-hoc test; n = 8) (Figure 10C). In comparison, no evident effects of Shp2 suppression on TUNEL apoptotic activation were observed in Cav-1^-/-^ mice group (Figure 10D).

Increased TUNEL staining was also observed in acute ocular hypertension model, in both WT and Cav-1^-/-^ mice groups although Cav-1^-/-^ retinas displayed less increase in TUNEL positive cells in GCL in glaucoma (*p < 0.05; one-way ANOVA; Bonferroni post-hoc test; n = 8) (Figure 10 F, G) when compared to the WT retinas (***p < 0.001; one-way ANOVA; Bonferroni post-hoc test; n = 8) (Figure 10 E, G). Interestingly, when WT and Cav-1^-/-^ mice were subjected to Shp2 KD in the acute model of increased IOP, no significant loss of TUNEL staining was observed.

The apoptotic pathway changes were further evaluated using cleaved caspase-3 (c-casp3) immunostaining under both chronic and acute experimental glaucoma conditions. Retinal imaging revealed that intensity of c-casp3 apoptotic marker staining in GCL was much higher in WT retinas following both chronic and acute models of elevated IOP when compared to Cav-1^-/-^ mice (****p < 0.0001; one-way ANOVA; n = 8) (Figure 10B, D, F, H). Parallel to the TUNEL staining results, Shp2 suppression under chronically elevated IOP resulted in significantly fewer caspase-3 positive cells in WT mice (****p < 0.0001, AAV-SC WT vs *p < 0.05, AAV-Shp2 KD WT; one-way ANOVA; Bonferroni post-hoc test; n = 8) (Figure 10 D). No significant differences in c-casp3 staining were observed in the Cav-1^-/-^ mice in response to Shp2 silencing under similar conditions (Figure 10). Caspase-3 immunoreactivity was further increased in both WT (**p < 0.01, one-way ANOVA; Bonferroni post-hoc test; n = 8) and Cav-1^-/-^ (*p < 0.05, one-way ANOVA; Bonferroni post-hoc test; n = 8) mice in AOH model similar to the TUNEL staining (Figure 10H), but Shp2 KD had no discernible protective effect in either of these groups. Together these results highlight the role of Cav-1 in mediating apoptotic activation as also previously reported in other cells 72, 73 and indicate that loss of this protein could efficiently hinder the induction of cellular apoptotic pathways under glaucomatous stress conditions. Moreover, the protective effects of Shp2 silencing in the RGCs are mediated through Cav-1 and accordingly Cav-1 ablated mice exhibited no protection upon Shp2 suppression in either of the RGC stress models.

### Downregulation of Shp2 prevents loss of integrin 1β/ FAK signalling in the retina in chronic glaucoma conditions

**Figure 11:**
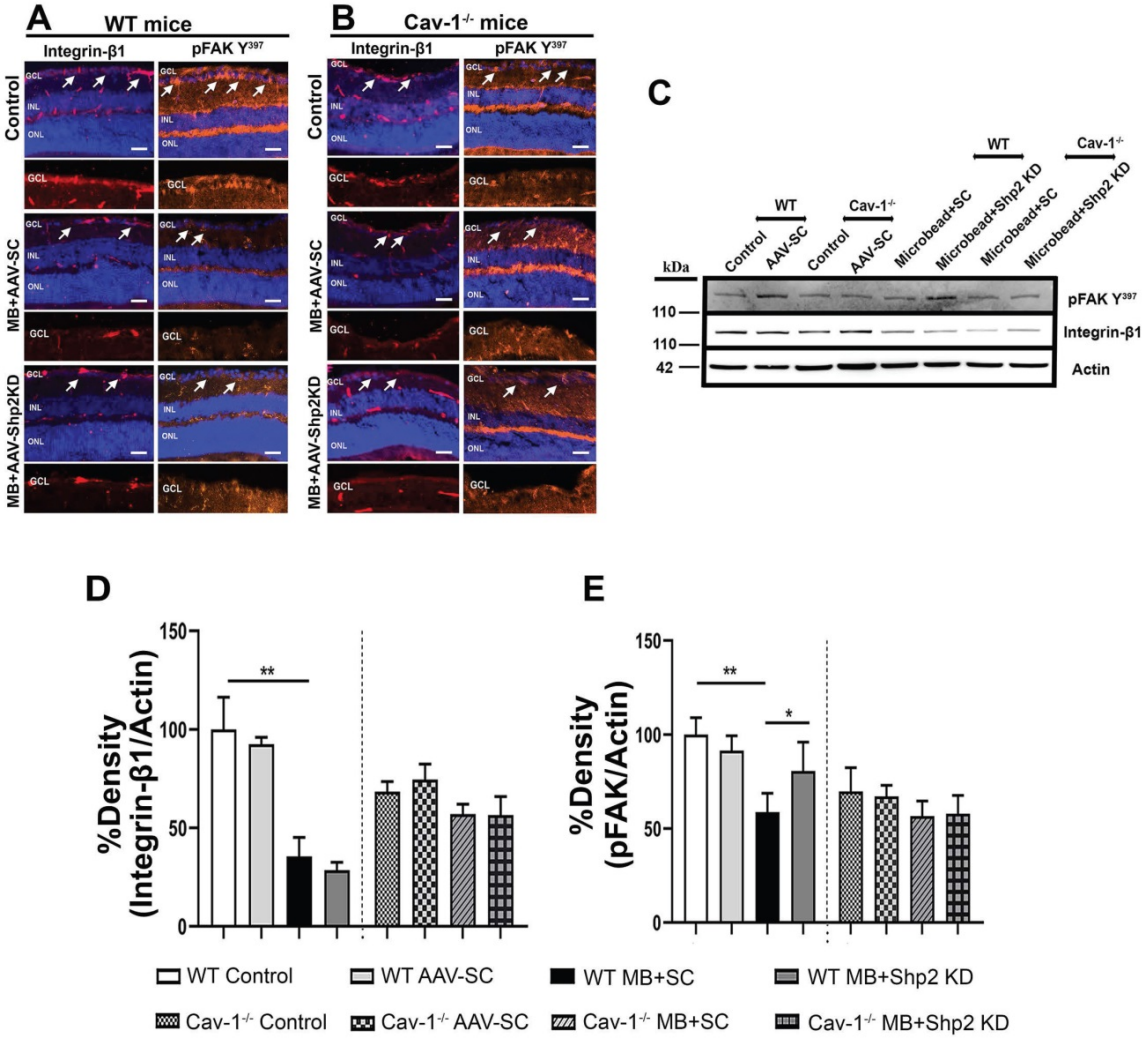

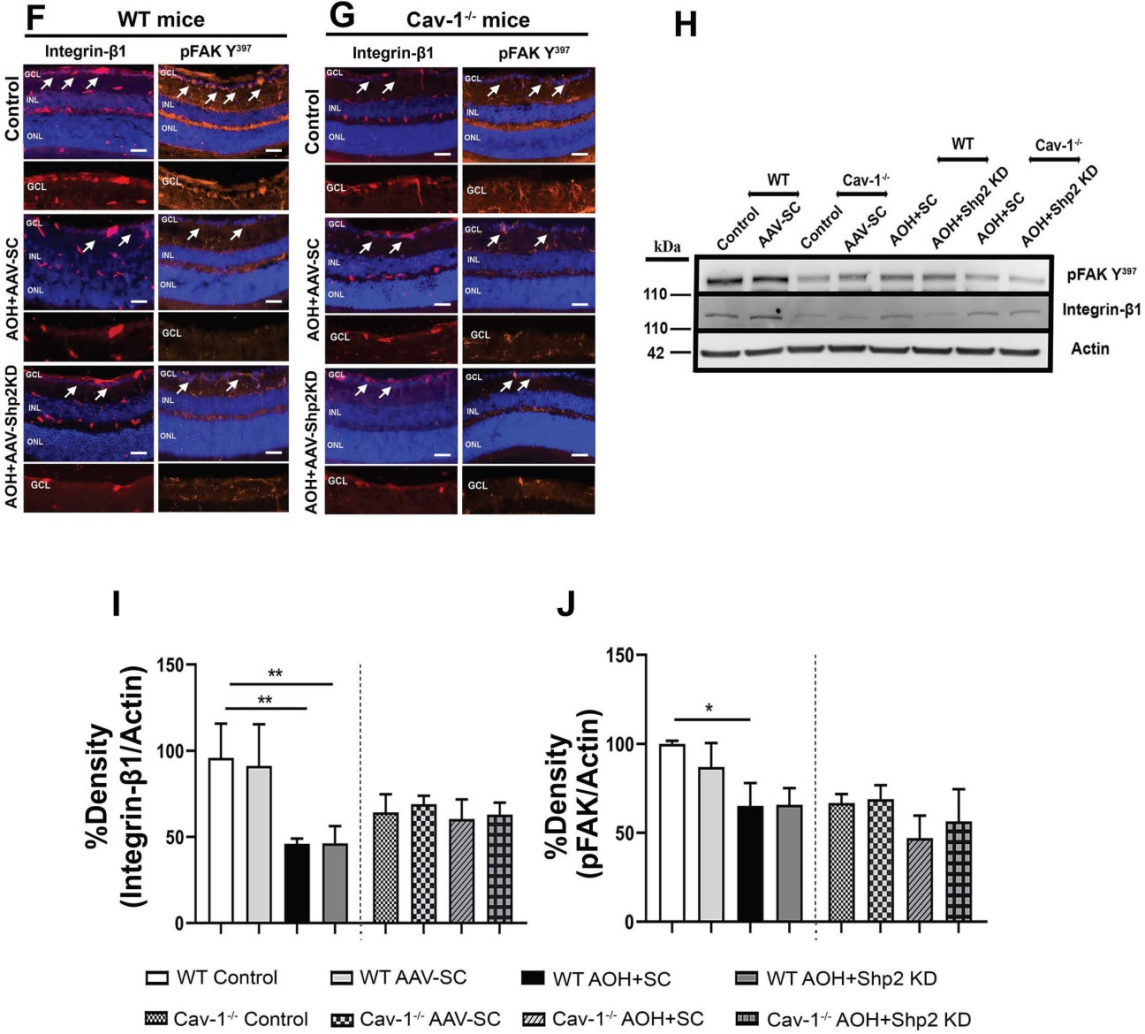
** Effects of Shp2 modulation on integrin β1/ FAK expression in WT and Cav-1^-/-^ retinas**. Immunofluorscence analysis of the retinal sections demonstrating integrin β1and its downstream effector FAK distribution within the retina in WT (A) and Cav-1 deficient (B) mice (Scale bar = 50 µm). (C) ONH lysates probed for integrin and phospho-FAK (Y^397^) using their specific antibodies with β-actin as loading control. (D, E) ANOVA Multiple analysis using Bonferroni post-hoc test revealed that a there was decline in integrin β1 (**p < 0.01, one-way ANOVA; Bonferroni post-hoc test; n = 6) and pFAK levels (**p < 0.01, one-way ANOVA; Bonferroni post-hoc test; n = 6) in WT retinas after exposure to chronic IOP stress. (E) Loss of FAK phosphorylation in WT animals was significantly protected upon subjecting retinas to AAV-Shp2 KD (*p < 0.05, one-way ANOVA; Bonferroni post-hoc test; n = 6). No significant differences were detected in the level of integrin β1 expression (D) or FAK activity (E) in Cav-1 deficient retinas under similar conditions. (F, G) Immunohistochemical images of the retinal sections stained integrin β1/ FAK expression following acute model of High IOP (Scale bar = 50 µm). (H-J) Immunoblotting and densitometric quantification indicated that similar to chronic type of high IOP, in acute IOP elevation there was significant decline in integrin β1 (**p < 0.01, one-way ANOVA; Bonferroni post-hoc test; n = 6) (I) and pFAK (*p < 0.05, one-way ANOVA; Bonferroni post-hoc test; n = 6) (J) in WT retinas compared to controls and Cav-1^-/-^ mice.

Recent evidence suggests that β1 integrin and its downstream effector protein focal adhesion kinase (FAK) play as an essential role in modulating RGC survival signalling and neurite growth 50,74. Shp2 phosphatase and Cav-1 are involved in regulating integrin signalling with Cav-1 being implicated in mediating integrin endocytosis through which it has been shown to modulate cell migration processes 75, 76. Shp2 has also been reported to play a role as an upstream regulator of FAK 77. Our results indicate that integrin/FAK pathway is disrupted in the retina in experimental glaucoma conditions in WT but not in Cav-1^-/-^ mice (Figure 11). Immunostaining with anti-β1 integrin and pFAK (Y^397^) identified that integrin β1 and its activated downstream intermediate, pFAK, were mainly localised to GCL and INL in retina (Figure 11). A much higher expression of β1 integrin receptor in WT retinas was observed in normal retina compared to the high IOP conditions and this expression was much increased in the WT compared to Cav-1^-/-^ animals (Figure 11 A, B/ F, G). WB analysis of the ONH lysates further validated these observations and established higher expression of both integrin β1 and pFAK Y^397^ markers in WT mice compared to the Cav-1 ablated retinas (Figure 11 C-E / H-J). A remarkable decline in both β1 Integrin (**p < 0.01, chronic; ** p < 0.01, acute high IOP; one-way ANOVA; Bonferroni post-hoc test; n = 6) and pFAK levels in WT retinas (**p < 0.01, chronic; * p < 0.05, acute high IOP; one-way ANOVA; Bonferroni post-hoc test; n = 6) was observed upon exposure to high IOP (Figure 11 I, J). No significant differences in the expression of these proteins were observed in Cav-1^-/-^ mice retinas. These observations highlight that Cav-1 may be involved in regulating integrin β1/ FAK signalling in the retina under both normal and glaucomatous stress conditions. We further investigated the effects of Shp2 silencing on the integrin 1β/ FAK signalling and noticed that AAV mediated Shp2 phosphatase silencing protected against the loss of FAK activity in chronically elevated IOP retinas (*p < 0.05, one-way ANOVA; Bonferroni post-hoc test; n = 6). This protection was not evident in Cav-1^-/-^ mice under similar conditions and was also not observed in the WT and Cav-1^-/-^ mice under acutely elevated IOP conditions. These observations suggest that protective effects of Shp2 silencing could potentially be mediated through its regulatory effects on FAK signalling in glaucoma. These results corresponded with previous observations indicating Shp2 role in regulating cell motility via FAK signalling pathways 78. Further, shp2 downregulation did not induce any alterations in integrin β1 expression in either chronic or acute IOP exposed retinal samples (Figure 11).

## Discussion

Shp2 has been demonstrated to regulate neurotrophin signaling pathways through its association with TrkA and TrkB tyrosine kinase receptor molecules 28, 29, 79. Neurotrophic factors, particularly BDNF and its high affinity receptor TrkB play critical roles in protecting retinal ganglion neurons during normal physiological conditions and under various forms of stress 80, 81. Previous research has implicated that Shp2 functions as a critical regulator of BDNF/TrkB survival signalling in the RGCs as well as in other neuronal cells 28, 29, 38, 39. Shp2 interaction with TrkB in the retinal neurons is mediated through the adaptor protein Cav-1 29. Cav1/ Cav2 gene locus variants have been reported to be associated with primary open angle glaucoma pathogenesis 46, 47. However, little is known about the functional and biochemical impact of Shp2 and Cav-1 interactions in the RGCs *in vivo* either under normal or stress conditions. Here we modulated Shp2 expression using intravitreal AAV administration and studied its impact on the retina using a combination of functional, biochemical and anatomical changes in the WT and Cav-1^-/-^ mice under normal physiological and experimental glaucoma conditions.

A major finding of this study was that while AAV-mediated Shp2 upregulation was detrimental to the inner retinal integrity, these effects were dependent on Cav-1. Moreover, results showed that genetic ablation of Cav-1 protein imparted protection against the inner retinal functional deficits in experimental glaucoma. Shp2 upregulation using AAV2 transduction resulted in blunting of pSTR amplitudes in WT retinas when compared to that of Cav-1^-/-^ mice (Figure 1 C). This specific loss of pSTR amplitudes in WT and not in Cav-1^-/-^ mice implied that Cav-1 ablation in retina was protective against Shp2 expression induced degenerative changes. This is in accordance with our previous observations in neuroblastoma cells where Shp2 upregulation resulted in cell growth inhibition while pharmacological inhibition of phosphatase using PHPS1 restored TrkB receptor activity and promoted neuronal cell survival 39, 82. Shp2 was also shown to negatively regulate TrkB, sensitizing the neurons to Ca^2+^-induced excitotoxicity damage whereas its deletion relieved TrkB and promoted neuronal cell survival 28.

In the retina, inner retinal electrophysiological observations correlated well with the biochemical and structural alterations with Shp2 upregulation resulting in loss of TrkB phosphorylation and thinning of GCL. Shp2 actions on TrkB have previously been suggested to be mediated through adapter protein Cav-1. Accordingly, we observed that Cav-1^-/-^ mice were protected against the loss of TrkB activation and GCL thinning induced by AAV induced Shp2 upregulation (Figure 2 and 3).

Further, we examined the effects of Shp2 silencing on functional, biochemical, and laminar structural aspects of the inner retina in WT and Cav-1 deficient mice under both chronic and acute experimental high IOP conditions. While Shp2 silencing rescued pSTR amplitude impairment observed in chronic IOP conditions in WT mice (Figure 4B), it did not protect the Cav-1^-/-^ retinas emphasizing that Shp2 effects are mediated through the Cav-1 (Figure 4B). A noticeable decline in TrkB phosphorylation (1.9 ± 0.6 fold) in GCL in WT but not in Cav-1^-/-^ retinas was observed following chronic IOP elevation (Figure 8A, B, F). This corresponded with an increased Cav-1 Y^14^ phosphorylation in chronically elevated IOP conditions (Figure S4). Shp2 encompasses an SH2 domain that obstructs its phosphatase activity under resting state 83, while its interactions with tyrosine-phosphorylated partners unlocks its auto-suppressing function 31, 84. Cav-1 protein is recognized for its adapter functions, and its phosphorylation contributes to SH2-domain-containing signalling intermediates, providing a docking site to recruit these motifs in the membrane thereby reconciling various signaling pathways 77, 85-87. Cav-1 may help anchor Shp2 SH2 motifs to the membrane microdomains 88, 89 and translocate a pool of phosphatase into the proximity of its substrate TrkB. The vicinity of two proteins and activated Shp2 phosphatase can then catalyze TrkB dephosphorylation and deactivate it. This increased Cav-1 phosphorylation in glaucomatous stress has previously been shown to enhance its interaction with the SH2 domain of Shp2 and relieve its autoinhibition 29. In addition, exposure to H_2_O_2_ induced oxidative stress manifests Shp2 phosphorylation in astrocytes and under these conditions Shp2 formed a complex with the Cav-1 protein 68, 90. Shp2 phosphatase was further shown to reciprocally co-immunoprecipitate with Cav-1 protein in RGCs under stress conditions 29. Taking that into consideration, Cav-1-mediated negative regulatory effects of Shp2 on TrkB activation might explain why direct BDNF/TrkB gene therapy has yielded only transient protection of RGCs in previous studies 80, 81, 91, 92. While BDNF/ TrkB activation is protective, simultaneous Shp2 activation in optic nerve injury models is likely to deactivate TrkB and limit beneficial effects of BDNF induced TrkB activation.

Correspondingly, the effects of Shp2 ablation on retina were also studied in an acute model of increased IOP 93, 94. Acute IOP increase resulted in significant diminution of pSTR amplitudes. Our findings were consistent with Kong et al. (2009) who demonstrated remarkably reduced pSTR amplitude at day 7 following elevated IOP exposure indicating the sensitivity of retinal ganglion neurons to spikes of IOP 95. However, in contrast to the chronic IOP model, inhibiting phosphatase expression did not impart significant protection against retinal functional and structural decline in either WT or Cav-1 null mice (Figure 5A-C). These data establish that inner retinal injury attributable to acute elevation of IOP is independent of Shp2 and Cav-1 effects. Intriguingly, loss of TrkB phosphorylation was evident in the acute IOP model indicating Shp2/ Cav-1 independent mechanisms that might be involved in regulating TrkB phosphorylation and establishing intrinsic differences in intracellular response to the two different categories of IOP stresses (Figure 9 F, G).

Both chronic and acute glaucoma models are shown to be associated with apoptotic activation in the inner retina. Shp2 silencing indeed resulted in reduced TUNEL positive cells in the retinas of WT mice in the chronic microbead model but no significant protective effects were evident in the acute IOP exposure model (Figure 10 A, E). Cav-1 null retinas although having a significant lower abundance of TUNEL positive cells under either of these glaucoma conditions, did not show any additional significant change in apoptotic activity upon Shp2 silencing (Figure 10 B, F). The apoptotic response was also investigated by probing for cleaved caspase-3 immunoreactivity. Widespread caspase 3 dependent apoptotic pathway activation has been described in the RGCs in experimental glaucoma conditions 96, 97. We discovered a marked increase in cleaved casp3 immunostaining in the GCL subsequent to IOP elevation in both the groups which was significantly reduced upon AAV-Shp2 shRNAmir administration. These results corroborated retinal TUNEL assay and histological observations that demonstrated decline in GCL density. Shp2 knock down has previously been shown to partially protect BDNF-treated cerebellar and SH-SY5Y neuronal cells against stress 28, 39 . Using Cav-1^-/-^ mice, here we have provided evidence that these anti-apoptotic effects of Shp2 silencing in chronic glaucoma conditions are dependent on Cav-1 protein. Cav-1 is also well expressed in other retinal cells such as Muller glial cells during development and in the adult retina. In this study, we have specifically modulated Shp2 expression in the ganglion cells using AAV, but it is possible that similar Shp2-Cav-1 interactions also occur in muller glial cells and other retinal neurons, which could indirectly impart protection to the ganglion cells.

Cav-1 has hitherto been shown to play pivotal role in maintaining integrin trafficking and regulating intracellular FAK signalling in myofibroblasts. Decline in Cav-1 expression was instrumental in loss of integrin endocytosis and disruption of ECM remodelling 75, 76. Integrin receptor family, particularly β1 isoform, is implicated in neuronal cell development, and cell migration, growth and motility through binding to extracellular matrix proteins and subsequent activation of various signalling pathways 52,98. Integrins are heterodimeric molecules that comprise α and β subunits with each type responding through interactions with different ECM ligands in signalling-specific manner 56. β1 integrin has specifically been implicated in regulating ganglion cell neurite outgrowth in the developing chick and mice retinas. β1 integrin and its major downstream intermediate tyrosine protein kinase FAK are ubiquitously expressed throughout the retina with distinctively high immunoreactivity in GCL 50,53,99. This study identified a significantly reduced expression of β1 integrin and FAK activity in Cav-1^-/-^ mice retinas (Figure 11). The results signify that Cav-1 is indispensable for regulation of integrin/FAK signalling pathway and accordingly ablation of Cav-1 led to negative regulation of this pathway in the RGCs (Figure 11 B, G). The findings advocate a key role of Cav-1 adaptor protein in helping to maintain integrin receptor expression and functional activity 75. A significant decline in both β1 integrin immunoreactivity and its downstream pFAK activation in GCL was more evident in WT upon IOP elevation than in Cav-1^-/-^ mice retinas (Figure 11A-J). These results substantiate prior findings by Santos et al (2012) that demonstrate loss of integrin, FAK and Akt activation in RGCs following retinal ischemia reperfusion injury. Laminin, which is one of the major ECM components was effective in rescuing RGCs under these conditions and promoted integrin signaling 50. Enhanced FAK activation was also shown to correlate with reduced apoptotic pathway activation in RGCs exposed to high IOP and loss of pFAK accompanied the exacerbation of RGC death 50,100. This study has made a novel observation that downregulation of Shp2 expression in GCL under chronic glaucomatous conditions could rescue integrin/FAK survival signalling as detected by enhanced FAK phosphorylation (Figure 11 C, E). No such protection was evident in Cav-1^-/-^ mice implying that Shp2 regulatory effects on pFAK signalling were mediated through the actions of Cav-1 adapter protein. The study has utilized global Cav-1^-/-^ mice in assessing retinal changes, which could have possible off-target effects such as through compromised blood retinal barrier and in this regard future investigations using ganglion cell specific conditional Cav-1^-/-^ mice will be valuable to understand disease mechanisms 101. Shp2 has previously been suggested to be an upstream regulator of FAK which synchronizes its dephosphorylation and was shown to induce attenuation of integrin-mediated Semaphorine-4D response in hippocampal neurons during development 102. Other reports have also suggested Shp2 as a critical component of integrin/ FAK signalling through which it stimulates a MAPK response 103,104. In summary, this study provides compelling evidence for the overarching role of Shp2-Cav-1 signaling axis in the inner retina in regulating TrkB and pFAK signaling as well as in preserving the integrity of inner retinal function and neuronal layers in chronic glaucoma conditions by suppressing apoptotic processes. We have provided molecular, functional and structural evidence that Shp2 upregulation in the inner retina is detrimental. However, Cav-1^-/-^ mice were more resistant to the unfavorable effects of an increase in phosphatase expression. In a similar manner, Shp2 silencing in chronic glaucoma model using AAV gene therapy was effective in protecting the retina in a Cav-1 dependent manner. These findings establish the molecular mechanisms underlying Shp2 role in glaucoma pathogenesis and highlight Shp2 as potential target for neuroprotective therapy in glaucoma and potentially other neurodegenerative disorders.

## Supplementary Material

Supplementary figures.

## Abbreviations

AOH: Acute ocular hypertension
BDNF: Brain derived neurotrophic factor
ERG: Electroretinogram
FAK: Focal adhesion kinase
GCL: Ganglion cell layer
H&E: Haematoxylin and eosin
INL: Inner nuclear layer
IOP: Intraocular pressure
IPL: Inner plexiform layer
pSTR: Positive Scotopic threshold response
RGCs: Retinal ganglion cells
Shp2: SH2 domain-containing tyrosine phosphatase-2
TrkB: Tropomyosin receptor kinase B

## References

[1] Casson RJ, Chidlow G, Wood JPMPM, Crowston JG, Goldberg I (2012). Definition of glaucoma: Clinical and experimental concepts. Clin Exp Ophthalmol.

[2] Cvenkel B, Kolko M (2020). Current Medical Therapy and Future Trends in the Management of Glaucoma Treatment. J Ophthalmol. 2020.

[3] Flaxman SR, Bourne RRA, Resnikoff S (2017). Global causes of blindness and distance vision impairment 1990-2020: a systematic review and meta-analysis. Lancet Glob Heal.

[4] Tham Y-CC, Li X, Wong TY, Quigley HA, Aung T, Cheng C-YY (2014). Global prevalence of glaucoma and projections of glaucoma burden through 2040: A systematic review and meta-analysis. Ophthalmology.

[5] Guymer C, Wood JP, Chidlow G, Casson RJ (2019). Neuroprotection in glaucoma: recent advances and clinical translation. Clin Experiment Ophthalmol.

[6] Kuehn MH, Fingert JH, Kwon YH (2005). Retinal ganglion cell death in glaucoma: Mechanisms and neuroprotective strategies. Ophthalmol Clin North Am.

[7] Munemasa Y, Kitaoka Y (2013). Molecular mechanisms of retinal ganglion cell degeneration in glaucoma and future prospects for cell body and axonal protection. Front Cell Neurosci.

[8] Mirzaei M, Pushpitha K, Deng L (2019). Upregulation of Proteolytic Pathways and Altered Protein Biosynthesis Underlie Retinal Pathology in a Mouse Model of Alzheimer's Disease. Mol Neurobiol.

[9] Peters D, Bengtsson B, Heijl A (2013). Lifetime Risk of Blindness in Open-Angle Glaucoma. Am J Ophthalmol.

[10] Deng L, Pushpitha K, Joseph C (2019). Amyloid β induces early changes in the ribosomal machinery, cytoskeletal organization and oxidative phosphorylation in retinal photoreceptor cells. Front Mol Neurosci.

[11] Quigley HA (2011). Glaucoma. In: The Lancet. Elsevier.

[12] Gupta N, Yücel YH (2007). Glaucoma as a neurodegenerative disease. Curr Opin Ophthalmol.

[13] Gupta VK, You Y, Gupta VB, Klistorner A, Graham SL (2013). TrkB receptor signalling: Implications in neurodegenerative, psychiatric and proliferative disorders [Internet]. Vol. 14, International Journal of Molecular Sciences. MDPI AG.

[14] Zhou Y, Pernet V, Hauswirth WW, Di Polo A (2005). Activation of the extracellular signal-regulated kinase 1/2 pathway by AAV gene transfer protects retinal ganglion cells in glaucoma. Mol Ther.

[15] Numakawa T, Suzuki S, Kumamaru E, Adachi N, Richards M, Kunugi H (2010). BDNF function and intracellular signaling in neurons. Histol Histopathol.

[16] Gupta V, You Y, Li J (2014). BDNF impairment is associated with age-related changes in the inner retina and exacerbates experimental glaucoma. Biochim Biophys Acta.

[17] Osborne A, Khatib TZ, Songra L (2018). Neuroprotection of retinal ganglion cells by a novel gene therapy construct that achieves sustained enhancement of brain-derived neurotrophic factor/tropomyosin-related kinase receptor-B signaling. Cell Death Dis.

[18] Ko ML, Hu DN, Ritch R, Sharma SC, Chen CF (2001). Patterns of retinal ganglion cell survival after brain-derived neurotrophic factor administration in hypertensive eyes of rats. Neurosci Lett.

[19] Wax MB, Tezel G (2002). Neurobiology of glaucomatous optic neuropathy: diverse cellular events in neurodegeneration and neuroprotection. Mol Neurobiol.

[20] Pernet V, Di Polo A (2006). Synergistic action of brain-derived neurotrophic factor and lens injury promotes retinal ganglion cell survival, but leads to optic nerve dystrophy *in vivo*. Brain.

[21] Dheer Y, Chitranshi N, Gupta VV (2018). Bexarotene Modulates Retinoid-X-Receptor Expression and Is Protective Against Neurotoxic Endoplasmic Reticulum Stress Response and Apoptotic Pathway Activation. Mol Neurobiol.

[22] Gupta VK, You Y, Li JC, Klistorner A, Graham SL (2013). Protective effects of 7,8-dihydroxyflavone on retinal ganglion and RGC-5 cells against excitotoxic and oxidative stress. J Mol Neurosci.

[23] Almasieh M, Wilson AM, Morquette B, Cueva Vargas JL, Di Polo A (2012). The molecular basis of retinal ganglion cell death in glaucoma. Prog Retin Eye Res.

[24] Fu QL, Li X, Yip HK (2009). Combined effect of brain-derived neurotrophic factor and LINGO-1 fusion protein on long-term survival of retinal ganglion cells in chronic glaucoma. Neuroscience.

[25] Vecino E, García-Grespo D, García M, Martinez-Millán L, Sharma SC, Carrascal E (2002). Rat retinal ganglion cells co-express brain derived neurotrophic factor (BDNF) and its receptor TrkB. Vision Res.

[26] Chitranshi N, Dheer Y, Abbasi M, You Y, Graham SL, Gupta V (2018). Glaucoma Pathogenesis and Neurotrophins: Focus on the Molecular and Genetic Basis for Therapeutic Prospects. Curr Neuropharmacol.

[27] Kumamaru E, Numakawa T, Adachi N, Kunugi H (2011). Glucocorticoid suppresses BDNF-stimulated MAPK/ERK pathway via inhibiting interaction of Shp2 with TrkB. FEBS Lett.

[28] Rusanescu G, Yang W, Bai A, Neel BG, Feig L a (2005). Tyrosine phosphatase SHP-2 is a mediator of activity-dependent neuronal excitotoxicity. EMBO J.

[29] Gupta VK, You Y, Klistorner A, Graham SL (2012). Shp-2 regulates the TrkB receptor activity in the retinal ganglion cells under glaucomatous stress. Biochim Biophys Acta - Mol Basis Dis.

[30] Abbasi M, Gupta V, Chitranshi N (2018). Regulation of Brain-Derived Neurotrophic Factor and Growth Factor Signaling Pathways by Tyrosine Phosphatase Shp2 in the Retina: A Brief Review. Front Cell Neurosci.

[31] Neel BG, Gu H, Pao L (2003). The 'Shp'ing news: SH2 domain-containing tyrosine phosphatases in cell signaling. Trends Biochem Sci.

[32] Tsang YH, Han X, Man WY, Lee N, Poon RYC (2012). Novel Functions of the Phosphatase SHP2 in the DNA Replication and Damage Checkpoints. Lee KS, Ed. PLoS One.

[33] Suzuki T, Matozaki T, Mizoguchi A, Kasuga M (1995). Localization and Subcellular Distribution of SH-PTP2, a Protein-Tyrosine-Phosphatase with Src Homology-2 Domains, in Rat Brain. Biochem Biophys Res Commun.

[34] Gómez del Rio MA, Sánchez-Reus MI, Iglesias I (2013). Neuroprotective Properties of Standardized Extracts of Hypericum perforatum on Rotenone Model of Parkinson's Disease. CNS Neurol Disord Drug Targets.

[35] Zhang X, Cai ZG, Simons DL (2011). Loss of Shp2-mediated mitogen-activated protein kinase signaling in muller glial cells results in retinal degeneration. Mol Cell Biol.

[36] Chong ZZ, Lin S-H, Kang J-Q, Maiese K (2003). The tyrosine phosphatase SHP2 modulates MAP kinase p38 and caspase 1 and 3 to foster neuronal survival. Cell Mol Neurobiol.

[37] Kinkl N, Hageman GS, Sahel JA, Hicks D (2002). Fibroblast growth factor receptor (FGFR) and candidate signaling molecule distribution within rat and human retina. Mol Vis.

[38] Chitranshi N, Dheer Y, Mirzaei M (2019). Loss of Shp2 Rescues BDNF/TrkB Signaling and Contributes to Improved Retinal Ganglion Cell Neuroprotection. Mol Ther.

[39] Chitranshi N, Dheer Y, Gupta VVV (2017). PTPN11 induces endoplasmic stress and apoptosis in SH-SY5Y cells. Neuroscience.

[40] Parton RG, del Pozo M a (2013). Caveolae as plasma membrane sensors, protectors and organizers. Nat Rev Mol Cell Biol.

[41] Surguchov A (2020). Caveolin: A New Link Between Diabetes and AD [Internet]. Vol. 40, Cellular and Molecular Neurobiology. Springer.

[42] Parton RG, Simons K (2007). The multiple faces of caveolae. Nat Rev Mol Cell Biol.

[43] Surgucheva I, Surguchov A Expression of caveolin in trabecular meshwork cells and its possible implication in pathogenesis of primary open angle glaucoma. 2011.

[44] Berta AI, Kiss AL, Kemeny-Beke A, Lukats A, Szabó A, Szél A (2007). Different caveolin isoforms in the retina of melanoma malignum affected human eye. Mol Vis.

[45] Gu X, Reagan A, Yen A, Bhatti F, Cohen AW, Elliott MH (2014). Spatial and temporal localization of caveolin-1 protein in the developing retina. Adv Exp Med Biol.

[46] Thorleifsson G, Walters GB, Hewitt AW (2010). Common variants near CAV1 and CAV2 are associated with primary open-angle glaucoma. Nat Genet.

[47] Wiggs JL, Kang JH, Yaspan BL (2011). Common variants near CAV1 and CAV2 are associated with primary open-angle glaucoma in Caucasians from the USA. Hum Mol Genet.

[48] Nunes HF, Ananina G, Costa VP, Zanchin NIT, de Vasconcellos JPC, de Melo MB (2018). Investigation of *CAV1/CAV2* rs4236601 and *CDKN2B-AS1* rs2157719 in primary open-angle glaucoma patients from Brazil. Ophthalmic Genet.

[49] Rong SS, Chen LJ, Leung CKS (2016). Ethnic specific association of the CAV1/CAV2 locus with primary open-angle glaucoma. Sci Rep.

[50] Santos ARC, Corredor RG, Obeso BA (2012). β1 Integrin-Focal Adhesion Kinase (FAK) Signaling Modulates Retinal Ganglion Cell (RGC) Survival. PLoS One.

[51] Morrison JC (2006). Integrins in the optic nerve head: potential roles in glaucomatous optic neuropathy (an American Ophthalmological Society thesis). Trans Am Ophthalmol Soc.

[52] Clegg DO (2000). Novel roles for integrins in the nervous system. Mol Cell Biol Res Commun.

[53] Vecino E, Heller JP, Veiga-Crespo P, Martin KR, Fawcett JW (2015). Influence of extracellular matrix components on the expression of integrins and regeneration of adult retinal ganglion cells. PLoS One.

[54] Cann GM, Bradshaw AD, Gervin DB, Hunter AW, Clegg DO (1996). Widespread Expression of β1 Integrins in the Developing Chick Retina: Evidence for a Role in Migration of Retinal Ganglion Cells. Dev Biol.

[55] Bradshaw AD, McNagny KM, Gervin DB, Cann GM, Graf T, Clegg DO (1995). Integrin alpha 2 beta 1 mediates interactions between developing embryonic retinal cells and collagen. Development.

[56] Vecino E, Rodriguez FD, Ruzafa N, Pereiro X, Sharma SC (2016). Glia-neuron interactions in the mammalian retina. Prog Retin Eye Res.

[57] Crowston JG, Kong YXG, Trounce IA (2015). An acute intraocular pressure challenge to assess retinal ganglion cell injury and recovery in the mouse. Exp Eye Res.

[58] Bui B V, Edmunds B, Cioffi GA, Fortune B (2005). The gradient of retinal functional changes during acute intraocular pressure elevation. Investig Ophthalmol Vis Sci.

[59] He Z, Bui B V, Vingrys AJ (2006). The rate of functional recovery from acute IOP elevation. Investig Ophthalmol Vis Sci.

[60] Lumayag S, Haldin CE, Corbett NJ (2013). Inactivation of the microRNA-183/96/182 cluster results in syndromic retinal degeneration. Proc Natl Acad Sci.

[61] You Y, Thie J, Klistorner A (2012). Normalization of visual evoked potentials using underlying electroencephalogram levels improves amplitude reproducibility in rats. Invest Ophthalmol Vis Sci.

[62] Dheer Y, Chitranshi N, Gupta VV (2019). Retinoid x receptor modulation protects against ER stress response and rescues glaucoma phenotypes in adult mice. Exp Neurol.

[63] You Y, Gupta VK, Li JC, Al-Adawy N, Klistorner A, Graham SL (2014). FTY720 protects retinal ganglion cells in experimental glaucoma. Invest Ophthalmol Vis Sci.

[64] Gupta V, Chitranshi N, You Y, Gupta V, Klistorner A, Graham S (2014). Brain derived neurotrophic factor is involved in the regulation of glycogen synthase kinase 3β (GSK3β) signalling. Biochem Biophys Res Commun.

[65] Gupta VK, Gowda LR (2008). Alpha-1-proteinase inhibitor is a heparin binding serpin: Molecular interactions with the Lys rich cluster of helix-F domain. Biochimie.

[66] Gupta VK, Rajala A, Rajala RVS (2012). Insulin receptor regulates photoreceptor CNG channel activity. Am J Physiol - Endocrinol Metab.

[67] Abbasi M, Gupta VK, Chitranshi N (2020). Caveolin-1 Ablation Imparts Partial Protection Against Inner Retinal Injury in Experimental Glaucoma and Reduces Apoptotic Activation. Mol Neurobiol.

[68] Park JS, Park SJ, Choi Y-H (2011). Caveolin-1 is involved in reactive oxygen species-induced SHP-2 activation in astrocytes. Exp Mol Med.

[69] Park Y-W, Jeong M-B, Lee ER (2013). Acute changes in central corneal thickness according to experimental adjustment of intraocular pressure in normal canine eyes. J Vet Med Sci.

[70] Nadal-Nicolás FM, Jiménez-López M, Salinas-Navarro M (2012). Whole Number, Distribution and Co-Expression of Brn3 Transcription Factors in Retinal Ganglion Cells of Adult Albino and Pigmented Rats. Harvey AR, Ed. PLoS One.

[71] Galindo-Romero C, Avilés-Trigueros M, Jiménez-López M (2011). Axotomy-induced retinal ganglion cell death in adult mice: Quantitative and topographic time course analyses. Exp Eye Res.

[72] Li Y, Luo J, Lau WM (2011). Caveolin-1 plays a crucial role in inhibiting neuronal differentiation of neural stem/progenitor cells via VEGF signaling-dependent pathway. PLoS One.

[73] Dasari A, Bartholomew JN, Volonte D, Galbiati F (2006). Oxidative stress induces premature senescence by stimulating caveolin-1 gene transcription through p38 mitogen-activated protein kinase/Sp1-mediated activation of two GC-rich promoter elements. Cancer Res.

[74] Goldberg JL, Espinosa JS, Xu Y, Davidson N, Kovacs GTA, Barres BA (2002). Retinal ganglion cells do not extend axons by default: promotion by neurotrophic signaling and electrical activity. Neuron.

[75] Shi F, Sottile J (2008). Caveolin-1-dependent 1 integrin endocytosis is a critical regulator of fibronectin turnover. J Cell Sci.

[76] Salanueva IJ, Cerezo A, Guadamillas MC, del Pozo MA (2007). Integrin regulation of caveolin function. J Cell Mol Med.

[77] Jo A, Park H, Lee SH (2014). SHP-2 binds to caveolin-1 and regulates Src activity via competitive inhibition of CSK in response to H2O2 in astrocytes. PLoS One.

[78] Inagaki K, Noguchi T, Matozaki T (2000). Roles for the protein tyrosine phosphatase SHP-2 in cytoskeletal organization, cell adhesion and cell migration revealed by overexpression of a dominant negative mutant. Oncogene.

[79] Goldsmith BA, Koizumi S (2002). Transient Association of the Phosphotyrosine Phosphatase SHP-2 with TrkA Is Induced by Nerve Growth Factor. J Neurochem.

[80] Chen H, Weber AJ (2001). BDNF enhances retinal ganglion cell survival in cats with optic nerve damage. Invest Ophthalmol Vis Sci.

[81] Cheng L, Sapieha P, Kittlerova P, Hauswirth WW, Di Polo A (2002). TrkB gene transfer protects retinal ganglion cells from axotomy-induced death *in vivo*. J Neurosci.

[82] Gupta V, Mirzaei M, Gupta VB (2017). Glaucoma is associated with plasmin proteolytic activation mediated through oxidative inactivation of neuroserpin. Sci Rep.

[83] Dance M, Montagner A, Salles JP, Yart A, Raynal P (2008). The molecular functions of Shp2 in the Ras/Mitogen-activated protein kinase (ERK1/2) pathway. Cell Signal.

[84] He R-J, Yu Z-H, Zhang R-Y, Zhang Z-Y (2014). Protein tyrosine phosphatases as potential therapeutic targets. Acta Pharmacol Sin.

[85] Park DS, Woodman SE, Schubert W (2002). Caveolin-1/3 double-knockout mice are viable, but lack both muscle and non-muscle caveolae, and develop a severe cardiomyopathic phenotype. Am J Pathol.

[86] Volonté D, Galbiati F, Pestell RG, Lisanti MP (2001). Cellular Stress Induces the Tyrosine Phosphorylation of Caveolin-1 (Tyr ^14^ ) via Activation of p38 Mitogen-activated Protein Kinase and c-Src kinase. J Biol Chem.

[87] Santilman V, Baran JA, Anand-Apte B, Evans RM, Parat MO (2007). Caveolin-1 polarization in transmigrating endothelial cells requires binding to intermediate filaments. Angiogenesis.

[88] Kim HY, Park EJ, Joe E -h, Jou I (2003). Curcumin Suppresses Janus Kinase-STAT Inflammatory Signaling through Activation of Src Homology 2 Domain-Containing Tyrosine Phosphatase 2 in Brain Microglia. J Immunol.

[89] Pawson T, Gish GD, Nash P (2001). SH2 domains, interaction modules and cellular wiring. Trends Cell Biol.

[90] Park H, Ahn KJ, Kang JL, Choi YH (2015). Protein-protein interaction between caveolin-1 and SHP-2 is dependent on the N-SH2 domain of SHP-2. BMB Rep.

[91] Martin KRG, Quigley HA, Zack DJ (2003). Gene therapy with brain-derived neurotrophic factor as a protection: retinal ganglion cells in a rat glaucoma model. Invest Ophthalmol Vis Sci.

[92] Wilson AM, Di Polo A (2012). Gene therapy for retinal ganglion cell neuroprotection in glaucoma. Gene Ther.

[93] Osborne NN, Wood JP, Chidlow G, Bae JH, Melena J, Nash MS (1999). Ganglion cell death in glaucoma: what do we really know?. Br J Ophthalmol.

[94] Chen B, Tang L (2011). Protective effects of catalase on retinal ischemia/reperfusion injury in rats. Exp Eye Res.

[95] Kong YX, Crowston JG, Vingrys AJ, Trounce IA, Bui B V (2009). Functional changes in the retina during and after acute intraocular pressure elevation in mice. Investig Ophthalmol Vis Sci.

[96] Kim HS, Park CK (2005). Retinal ganglion cell death is delayed by activation of retinal intrinsic cell survival program. Brain Res.

[97] Tchedre KT, Yorio T (2008). σ-1 Receptors Protect RGC-5 Cells from Apoptosis by Regulating Intracellular Calcium, Bax Levels, and Caspase-3 Activation. Investig Opthalmology Vis Sci.

[98] Brakebusch C, Fässler R (2003). NEW EMBO MEMBER'S REVIEW: The integrin-actin connection, an eternal love affair. EMBO J.

[99] Gupta VK, Rajala A, Daly RJ, Rajala RVS (2010). Growth factor receptor-bound protein 14: a new modulator of photoreceptor-specific cyclic-nucleotide-gated channel. EMBO Rep.

[100] Gibson RM, Craig SE, Heenan L, Tournier C, Humphries MJ (2005). Activation of integrin α5β1 delays apoptosis of Ntera2 neuronal cells. Mol Cell Neurosci.

[101] Trushina E, Du Charme J, Parisi J, McMurray CT (2006). Neurological abnormalities in caveolin-1 knock out mice. Behav Brain Res.

[102] Fuchikawa T, Nakamura F, Fukuda N, Takei K, Goshima Y (2009). Biochemical and Biophysical Research Communications Protein tyrosine phosphatase SHP2 is involved in Semaphorin 4D-induced axon repulsion. Biochem Biophys Res Commun.

[103] Oh ES, Gu H, Saxton TM (1999). Regulation of early events in integrin signaling by protein tyrosine phosphatase SHP-2. Mol Cell Biol.

[104] Oh E-SS, Gu H, Saxton TM (2015). Regulation of Early Events in Integrin Signaling by Protein Tyrosine Phosphatase SHP-2. Mol Cell Biol.

