# Supplementary material for: Inner retinal injury in experimental glaucoma is prevented upon AAV mediated Shp2 silencing in a caveolin dependent manner
Source: Theranostics. 2021 Apr 15;11(13):6154–72. doi: 10.7150/thno.55472 (PMC8120201; doi:10.7150/thno.55472)
Supplement: Supplementary file 1 — Supplementary figures. [file thnov11p6154s1.pdf]

Additional file: Supplementary methods, results and figures:

AAV2 gene therapy to modulate Shp2 expression in the GCL

Mice were subjected to intravitreal delivery of AAV transgene expression cassettes encoding eGFP and Shp2 (Figure S1A) or small hairpin RNA (shRNA)mir for Shp2 and control scrambled sequence (Figure S1B) leading to AAV-mediated Shp2 overexpression and downregulation respectively. eGFP sequence within Shp2 expression cassettes was used to trace the AAV expression in RGCs. Retinal sections were probed for alterations in eGFP and Shp2 expression in all group types at the end of specified time points. Using immunofluorescence assay (IF), we observed a significantly increased Shp2 immunoreactivity in AAV-Shp2 administered retinas, while in the retinas transduced with AAV2-Shp2 shRNAmir vectors, phosphatase immunoreactivity was significantly reduced compared to AAV-scrambled controls (Figure S1C).

A high GFP expression was observed in AAV-GFP, AAV-Shp2 and AAV-shRNAmir groups two months post-injection reflecting a high efficiency of AAV2 mediated gene delivery to the GCL in mice retinas (Figure S1D). The expression of Shp2 and eGFP were primarily localized to the retinal ganglion cell layer (Figure 1C,D) and confirmed through subjecting the retinas to specific RGC marker, Brn3a staining (Nadal-Nicola´s et al., 2009; Galindo-Romero et al., 2011; Nadal-Nicolás et al., 2012) which co-expressed along with eGFP and Shp2 in the GCL.

Figure S1

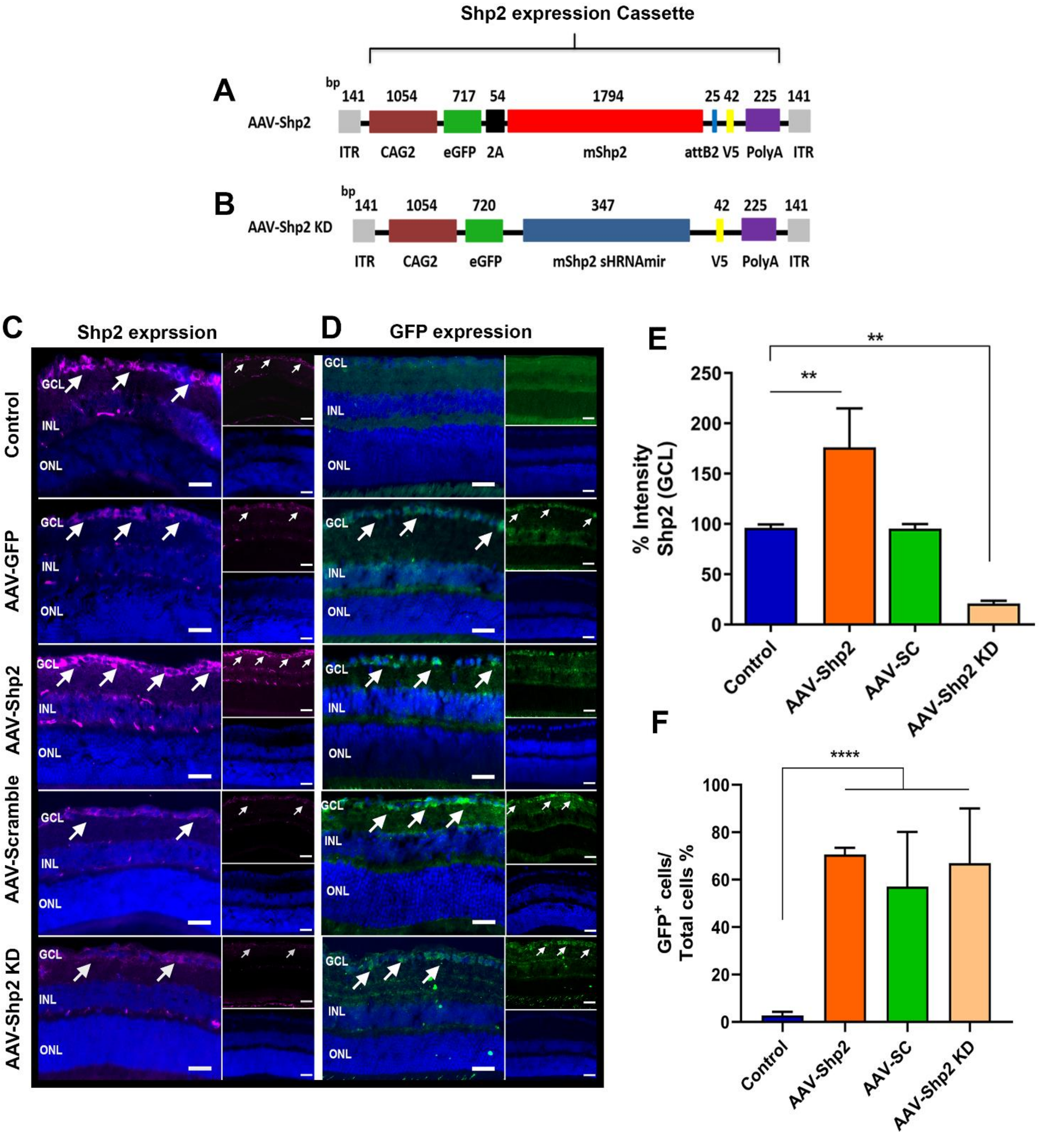

Shp2 expression Cassette
A
bp
AAV-Shp2
141 1054 717 54 1794 25 42 225 141
ITR CAG2 eGFP 2A mShp2 attB2 V5 PolyA ITR
B
bp
AAV-Shp2 KD
141 1054 720 347 42 225 141
ITR CAG2 eGFP mShp2 sHRNAmir V5 PolyA ITR
C
Shp2 exprssion
D
GFP expression
Control
AAV-GFP
AAV-Shp2
AAV-Scramble
AAV-Shp2 KD
GCL
INL
ONL
E
% Intensity Shp2 (GCL)
0
50
100
150
200
250
**
**
Control
AAV-Shp2
AAV-SC
AAV-Shp2 KD
F
GFP+ cells/ Total cells %
0
20
40
60
80
100
****
Control
AAV-Shp2
AAV-SC
AAV-Shp2 KD

Figure S1: AAV-mediated Modulation of Shp2 expression in GCL. *(A, B) Schematic representation of the AAV vector design map with CAG hybrid promoter. AAV vector contained GFP, mShp2, mShp2 shRNmir and scrambled shRNA sequences (B) used for either Shp2 overexpression or its knockdown respectively. Vector also contained a T2A self-cleaving peptide sequence. Other regions included: Ori, origin of replication; CAG2 promoter, CMV, i.e. enhancer, cytomegalovirus immediate-early enhancer/chicken β-actin; GFP, green fluorescent protein; mSHP2, mouse Shp2, bGH poly(A), bovine growth hormone polyA sequence; ITR, inverted terminal repeat. (C) Two months following intravitreal injection of AAV, mice were sacrificed, and retinal sections immunostained with anti-Shp2 (purple), anti-GFP (green) and DAPI (blue) to evaluate retinal expression changes. The Right end panel shows DAPI staining to delineate different retinal layers Scale bar = 50 µm. (E, F) Comparison of the intensity of GFP and Shp2 expression. Significant expression of GFP observed in different groups compared to non-injected control eyes (**$p < 0.001$ one-way ANOVA; $n = 6$). A statistically significant upregulation of Shp2 (**$p < 0.001$; one-way ANOVA; $n = 6$) and its downregulation (**$p < 0.001$; one-way ANOVA; $n = 6$) following intravitreal administration of AAV-Shp2 and AAV-Shp2 KD construct respectively when compared to corresponding GFP and AAV-Scrambled (AAV-SC) controls.*

Figure S2

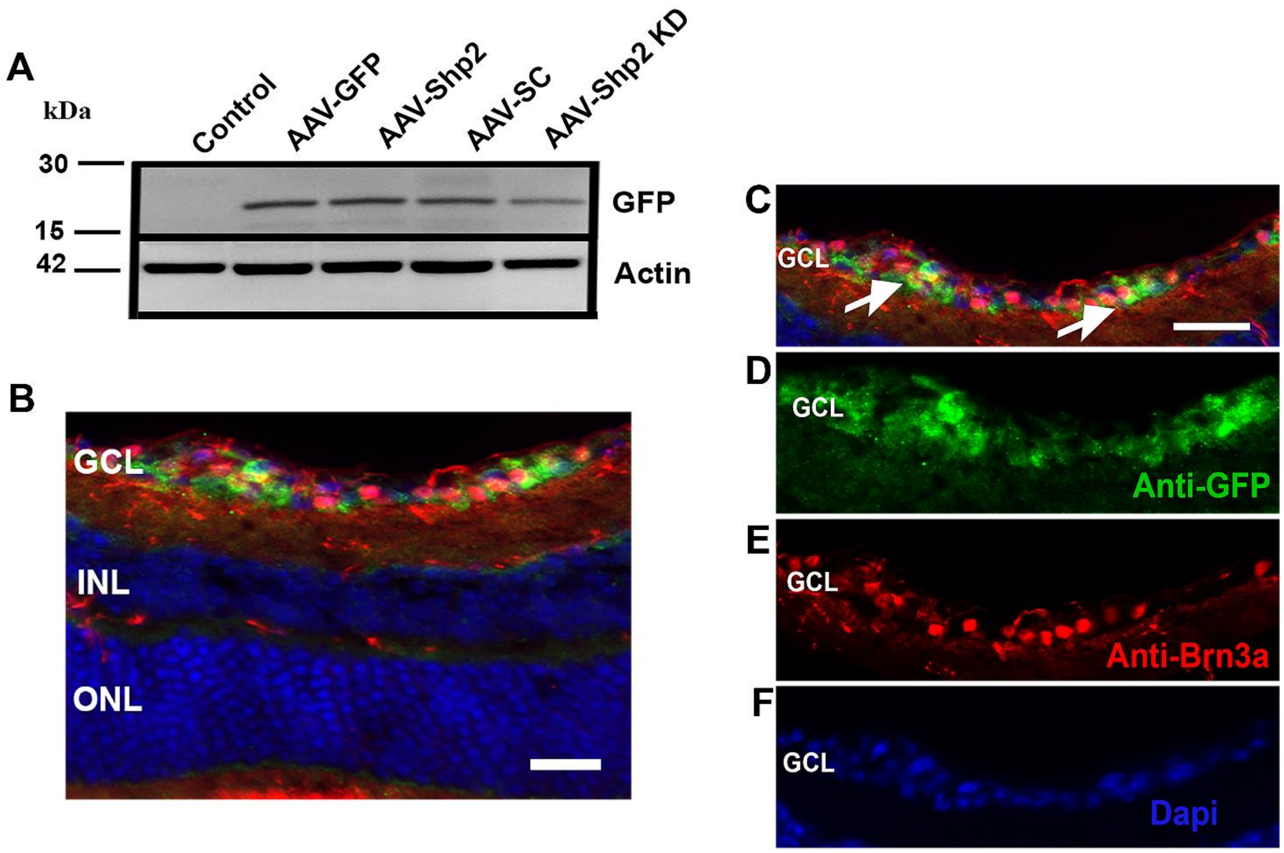

Figure S2: *(A) Western blot analysis indicating GFP expression in ONH lysate of retinas including controls as well as retinas subjected to AAV-GFP, AAV-Shp2, AAV-scramble and AAV-Shp2shRNAmir transduction. (B) Immunofluorescence image of mouse retina (scale bar = 50 µm) illustrating double immunostaining with GFP and Brn3a a specific RGC marker, two months following viral injection (C) shows co-localization of GFP labelled RGCs and Brn3a positive ganglion cells. Other images show cells labelled with GFP (D), Brn3a (E) as well as nuclei which are labelled with DAPI (F). Scale bar = 50µm. GCL, ganglion cell layer; INL, inner nuclear layer; ONL, outer nuclear layer.*

Figure S3

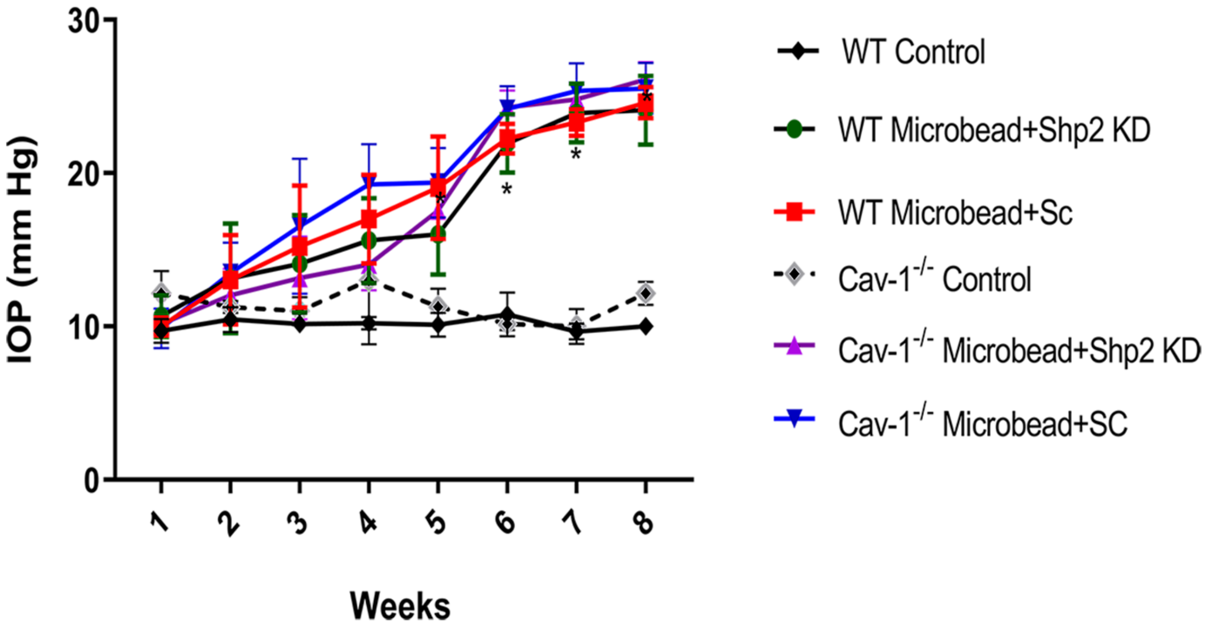

Figure S3: Alterations in IOP in both WT and Cav-1$^{-/-}$ eyes following weekly microbead injections for two months*. The IOP elevation injection in mice eyes ranged from 24.10 ± 5.17 mmHg and 26.12 ± 6.40 mmHg in WT and Cav-1$^{-/-}$ experimental groups and 10.78 ± 0.36 mmHg and 12 ± 1.02 mmHg for their controls respectively.*

Figure S4

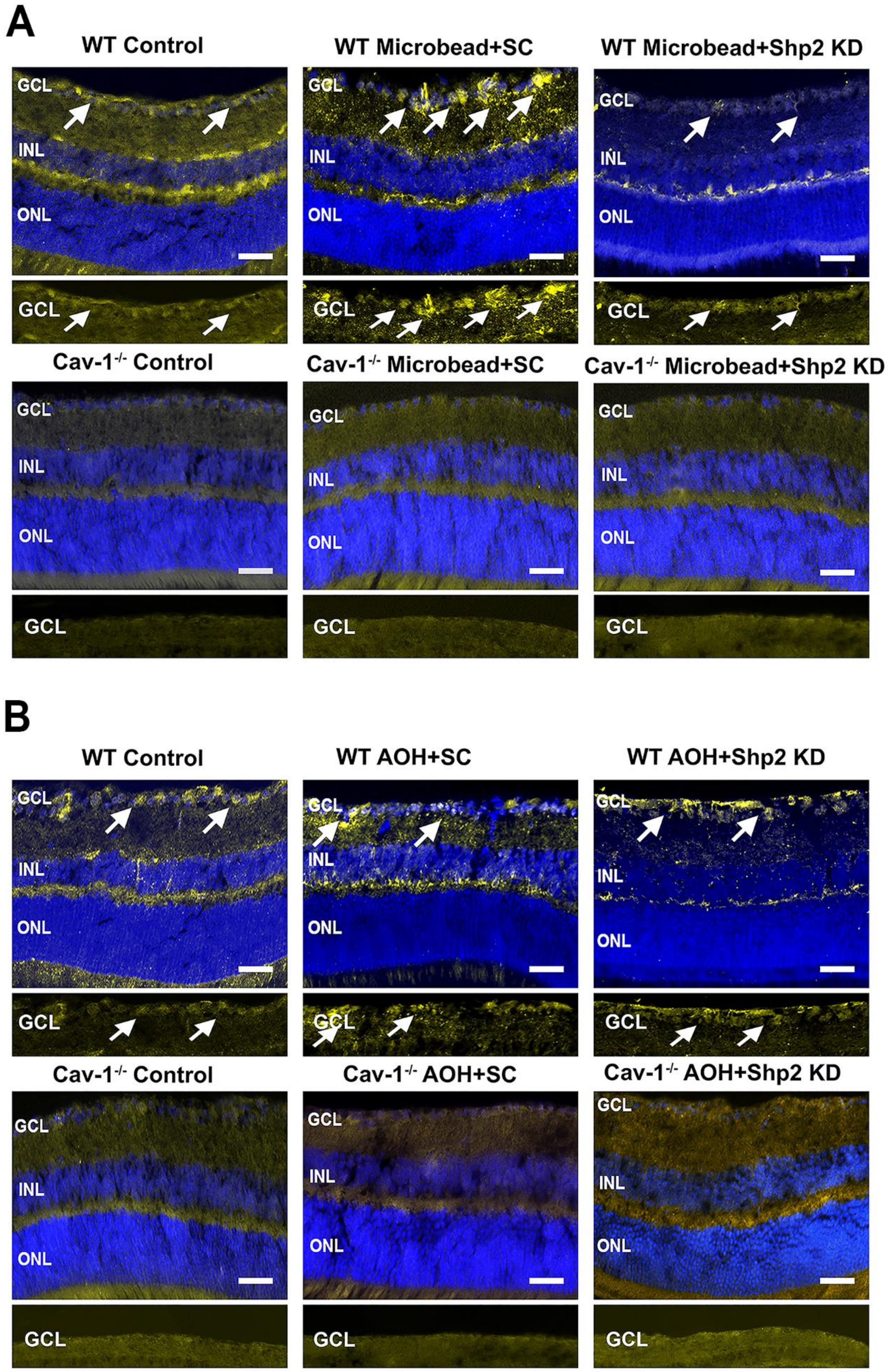
A
WT Control
WT Microbead+SC
WT Microbead+Shp2 KD
GCL
INL
ONL
Cav-1$^{-/-}$ Control
Cav-1$^{-/-}$ Microbead+SC
Cav-1$^{-/-}$ Microbead+Shp2 KD
B
WT Control
WT AOH+SC
WT AOH+Shp2 KD
Cav-1$^{-/-}$ Control
Cav-1$^{-/-}$ AOH+SC
Cav-1$^{-/-}$ AOH+Shp2 KD

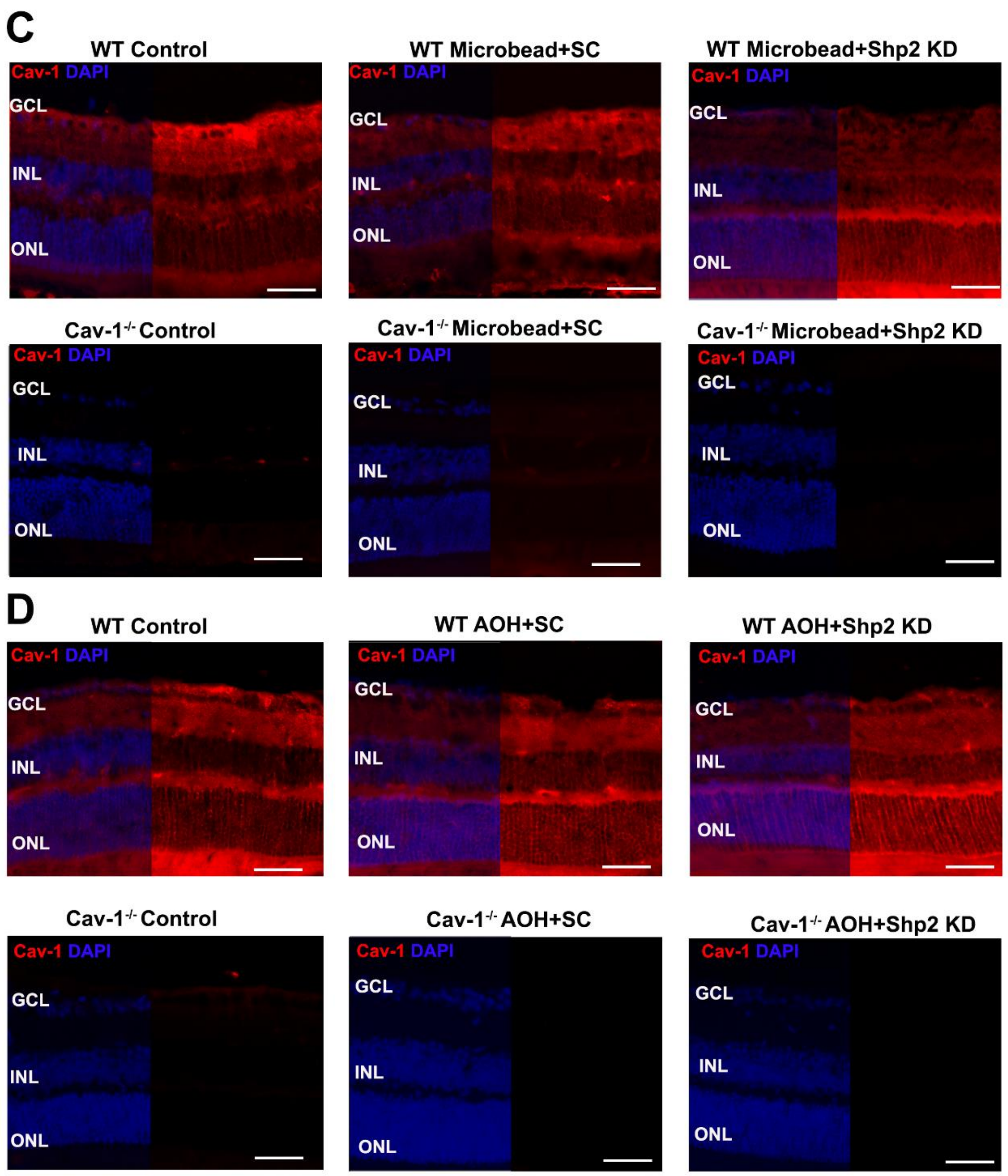

Figure S4: Cav-1 phosphorylation increased following elevated IOP in WT GCL*. (A) Evaluating pCav-1 Y14 in the retina 2 months following microbead injection revealed a noticeable increase in Cav-1 phosphorylation in WT group exposed to chronic high IOP and it significantly decreased following Shp2 silencing. No significant changes were observed in Cav-1*$^{-/-}$ *group (B) No remarkable changes occurred in Cav-1 phosphorylation either prior or subsequent to Shp2 downregulation in acute high IOP model in WT or Cav-1*$^{-/-}$ *groups. (C, D) There was no expression of Cav-1 in retinal layers of Cav-1 knockout mice. Additionally,*

immunofluorescence staining of the retinas against Cav-1 did not reveal any significant changes in the expression of this protein either prior or after 2 models of experimental glaucoma.
